# Systematic review and meta-analysis of economic and healthcare resource utilization outcomes for robotic versus manual total knee arthroplasty

**DOI:** 10.1007/s11701-023-01703-x

**Published:** 2023-10-11

**Authors:** Daniel Hoeffel, Laura Goldstein, Dhara Intwala, Lisa Kaindl, Aidan Dineen, Leena Patel, Robert Mayle

**Affiliations:** 1 DePuy Synthes, Raynham, MA USA; 2 EVERSANA, Burlington, ON Canada; 3 California Pacific Orthopaedics, San Francisco, CA USA

**Keywords:** Total knee arthroplasty, Robotic, Systematic literature review, Meta-analysis, Healthcare resource use, Health economics

## Abstract

The introduction of robotics in orthopedic surgery has led to improved precision and standardization in total knee arthroplasty (TKA). Clinical benefits of robotic versus manual TKA have been well established; however, evidence for economic and healthcare resource utilization outcomes (HRU) is lacking. The primary objective of this study was to compare economic and HRU outcomes for robotic and manual TKA. The secondary objective was to explore comparative robotic and manual TKA pain and opioid consumption outcomes. Multi-database literature searches were performed to identify studies comparing robotic and manual TKA from 2016 to 2022 and meta-analyses were conducted. This review included 50 studies with meta-analyses conducted on 35. Compared with manual TKA, robotic TKA was associated with a: 14% reduction in hospital length of stay (*P* = 0.022); 74% greater likelihood to be discharged to home (*P* < 0.001); and 17% lower likelihood to experience a 90-day readmission (*P* = 0.043). Robotic TKA was associated with longer mean operating times (incision to closure definition: 9.27 min longer, *P* = 0.030; general operating time definition: 18.05 min longer, *P* = 0.006). No differences were observed for total procedure cost and 90-day emergency room visits. Most studies reported similar outcomes for robotic and manual TKA regarding pain and opioid use. Coupled with the clinical benefits of robotic TKA, the economic impact of using robotics may contribute to hospitals’ quality improvement and financial sustainability. Further research and more randomized controlled trials are needed to effectively quantify the benefits of robotic relative to manual TKA.

**Supplementary Information:**

The online version contains supplementary material available at 10.1007/s11701-023-01703-x.

## Introduction

Osteoarthritis affects more than 25% of the adult population in the United States (US) [1]. The knee is the joint most commonly impacted by osteoarthritis [2]. Patients with knee osteoarthritis experience progressive pain and joint damage, functional disability, and an overall decline in quality of life [3]. In addition, patients experience an increased incidence of comorbidities and excess mortality, which is likely influenced by their reduced ability to perform physical activity [3]. Knee osteoarthritis can create an economic burden leading to wage losses and substantial direct medical costs for patients [3, 4]. With population growth, aging, and the obesity epidemic, the health and economic burden of osteoarthritis is expected to rise further [2].

Total knee arthroplasty (TKA) is a common and effective treatment for knee osteoarthritis that reduces pain and improves knee function in patients with advanced disease [5]. The surgery is personalized for each patient, with the most common aim of achieving neutral alignment of the lower limb and soft tissue balance of the knee [6]. Over the next 10 years, it is projected that the total annual use of TKA will increase by 80% in the US with an estimated 1.9 million patients undergoing the procedure annually [7].

Robotic TKA is a relatively new technology developed to increase precision and standardization compared to manual surgery [8–10]. Since 2010, interest and utilization of robotic TKA continue to increase across the US, with expected continued growth and adoption [11, 12]. Compared with manual TKA, robotic TKA has been shown to improve limb alignment, implant position, and functional outcomes [8–10, 13, 14].

While several systematic literature reviews (SLRs) and meta-analyses comparing robotic and manual TKA have been published, the focus has generally been on clinical and functional outcomes [10, 13–22]. The clinical value of robotic TKA has been well established, however, the benefits of increased precision and standardization of robotic TKA are accompanied by incremental costs for healthcare providers. Hospitals are faced with increased expenditures due to the cost of the robotic capital equipment as well as additional disposables that are required for each case [23]. We conducted an SLR and meta-analysis to better understand the impact of robotic TKA on economic and healthcare resource utilization (HRU) outcomes to determine whether the incremental cost of capital and disposables is offset by improvements in other healthcare outcomes.

The primary objective of this study was to compare economic and HRU outcomes for robotic vs. manual TKA using studies published in the last five years. Specifically, the primary objective was to address the evidence gap in the literature by conducting meta-analyses for procedure cost, operating time, hospital length of stay (LOS), odds of being discharged to home, odds of 90-day readmission, and odds of 90-day emergency room (ER) visit. The secondary objective of this study was to explore comparative robotic vs. manual TKA pain outcomes and patient opioid consumption reported in the literature.

## Methods

### Literature search

The goal of the literature search was to identify studies published from January 1, 2016 to January 2022 that were comparative for robotic vs. manual TKA and, if feasible, conduct meta-analyses for the outcomes of interest. An initial literature search was conducted on September 26, 2019 to identify studies published from January 2000 to September 2019 and an updated search was performed on January 14, 2022 to identify studies published after September 2019. Studies included in this review and meta-analysis were limited to those published in the last five years to reduce bias from older studies that may not align with current clinical practices.

The SLRs were performed according to the Preferred Reporting Items for Systematic reviews and Meta-Analyses (PRISMA) criteria [24]. In the initial search, EMBASE, MEDLINE, EBM Reviews, and EconLit databases were searched via the OVID platform, and the first three databases were searched in the update. The EconLit database was not searched in the update based on the rationale that coverage in this database is focused primarily on general economic and business journals rather than journals publishing on health economics. Studies were selected according to pre-specified criteria using the PICOS framework (see supplemental material). Bibliographies of included studies in identified SLRs and studies found on robot manufacturer websites were hand-searched for additional eligible studies. Two reviewers independently screened titles and abstracts of all identified records, then independently assessed the full texts of potentially relevant articles. Conflicts were resolved by a third reviewer. The full electronic search strategies and PICOS are found in the supplemental material. Studies were included here if they reported at least one outcome that compared robotic to manual TKA. Key study information, baseline characteristics, and outcomes measured from included studies were extracted into a dedicated data extraction form.

### Quality assessment

Quality of included studies was assessed using the appropriate tools [25]: randomized controlled trials with the NICE Single Technology Appraisal Evidence Submission Checklist [26]; non-randomized cohort studies with the Newcastle–Ottawa Non-randomized Cohort Study Tool [27]; and non-randomized case–control studies with the Newcastle–Ottawa Non-randomized Case–Control Study Tool [27].

### Statistical analysis

Meta-analyses were performed on summary level data for each outcome of interest: procedure cost, operating time, hospital LOS, discharge to home, 90-day readmissions, and 90-day ER visits. Outcome data from included studies were summarized using descriptive statistics (e.g., mean and standard deviation [SD] for continuous variables, and number and proportion for categorical variables). All costs were inflated and/or converted to 2022 US dollars to maximize comparability [28]. Random-effects models were used to take into consideration variations between studies and forest plots were generated. Summary statistics were presented as either ratio of means (RoM), weighted mean differences (WMD), or odds ratios (OR) and the corresponding 95% confidence interval (CI) was calculated for each variable. The percentage of total variation across studies was represented by *I*^*2*^*.* All analyses were conducted using R metafor package version 3.0–2 [29].

## Results

### Systematic literature review

**Fig. 1:**
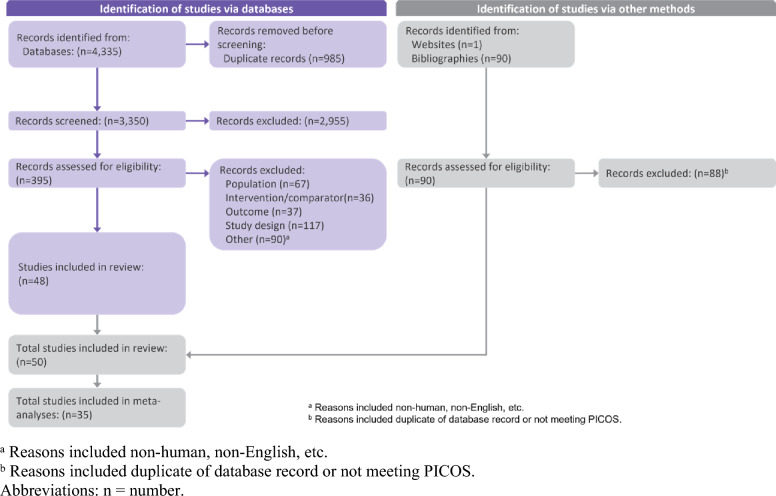
PRISMA flow diagram

A total of 4335 database records (3754 records from the initial database search, and an additional 581 records from the updated database search) were identified. Of the 3350 unique records, 2,955 were excluded during the title and abstract review and 395 were assessed at the full text level, where 347 were excluded. Additional two studies identified through hand-searching were included for a total of 50 studies from all searches included in this review. When selecting studies for the analyses, those that did not report adequate data (i.e., SDs, sample sizes) for the outcomes of interest were excluded for a total of 35 primary studies included in the meta-analyses (Table 1). The PRISMA flow diagram is provided in Fig. 1 (see supplemental material for a list of all 50 studies included in the systematic review).

**Table 1:** Studies included in meta-analyses

Study	Country	Robotic TKA *N*^a^	Manual TKA *N*^a^	Procedure cost	Operating time	Length of stay	% Discharged home	90-Day readmissions	90-Day ER visits
Archer (2021) [30]	US	4303	5993	–	–	Yes	Yes	–	–
Bendich (2021) [11]	US	4658	1,213,038	–	–	Yes	–	Yes	–
Bollars (2020) [31]	Belgium	77	77	–	Yes	–	–	–	–
Cool (2019) [32]	US	519	2595	–	–	–	Yes	Yes	–
Cotter (2020) [33]	US	147^b^	139^b^	Yes	Yes	Yes	Yes	Yes	Yes
Emara (2021) [34]	US	72,916	292,896	Yes	–	Yes	Yes	–	–
Fang (2022) [23]	US	263	1795	–	Yes	Yes	Yes	–	–
Grosso (2020) [35]	US	460	460	Yes	–	Yes	–	Yes	Yes
Hamilton (2021) [36]	US	83	83	–	–	–	Yes	–	–
Held (2021) [37]	US	37	49	–	Yes	–	–	–	–
Jeon (2019) [38]	Korea	84^b^	79^b^	–	Yes	–	–	–	–
Kaneko (2021) [39]	Japan	41	41	–	Yes	–	–	–	–
Kayani (2018) [40]	UK	40	40	–	Yes	–	–	–	–
Kayani (2019) [41]	UK	60	60	–	Yes	–	–	–	–
Kayani (2021) [42]	UK	15	15	–	Yes	–	–	–	–
Kim (2020) [43]	Korea	724^b^	724^b^	–	Yes	–	–	–	–
King (2020) [44]	US	202	290	–	–	–	–	Yes	–
Liow (2017) [45]	Singapore	31	29	–	Yes	Yes	–	–	–
Marchand (2017) [46]	US	20	20	–	Yes	–	–	–	–
Marchand (2020) [47]	US	140	60	–	Yes	–	–	–	–
Mitchell (2021) [48]	US	148^b^	139^b^	–	–	–	Yes	Yes	–
Mont (2019) [49]	US	519	2595	–	–	–	–	–	Yes
Ofa (2020) [50]	US	750,122	5228	–	–	Yes	–	Yes	
Pierce (2020) [51]	US	357	1785	–	–	–	–	–	Yes
Samuel (2021) [52]	US	85	255	–	–	Yes	Yes	Yes	Yes
Savov (2021) [53]	Germany	70	70	–	Yes	–	–	–	–
Shah (2021) [54]	US	4351	194,020	–	–	Yes	Yes	Yes	–
Shaw (2021) [55]	US	260	900	–	Yes	Yes	–	Yes	Yes
Sodhi (2018) [56]	US	240	40	–	Yes	–	–	–	–
Steffens (2021) [57]	Australia	77	181	Yes	Yes	Yes	Yes	–	–
Thiengwittayaporn (2021) [58]	Thailand	75	77	–	Yes	–	–	–	–
Tompkins (2021) [59]	US	2392	2392	–	–	Yes	Yes	–	–
Vanlommel (2021) [60]	Belgium	90	90	–	Yes	–	–	–	–
Zak (2021) [61]	US	101	217	–	Yes	–	–	–	–
Zhang (2021) [62]	US	120	120	–	Yes	Yes	Yes	–	–

^a^*N* represents the number of patients unless otherwise specified

^b^*N* represents number of knees, rather than number of patients

Abbreviations: *ER* emergency room; *N* number; *TKA* total knee arthroplasty; *UK* United Kingdom; *US* United States

### Quality assessment

Of the 50 included studies, 4 RCTs, 43 non-randomized comparative cohort studies, and 3 non-randomized comparative case–control studies were assessed with the instruments described in the methods. Overall, the four RCTs were assessed to be of reasonable to good quality [42, 43, 45, 58]. Randomization of participants was appropriate in all studies, with no unexpected imbalances and no evidence of reporting bias. Prognostic factors between groups were similar in three studies and this was unclear in one [45]. In two studies, care providers and participants were blinded to treatment allocation [42, 58]. Of the 43 non-randomized cohort studies, 41 were deemed to be of good to high quality (score of 7–9) and two were found to be of lower quality (score < 7) [63, 64]. These two studies did not contribute to any of the meta-analyses reported here, but were included in the narrative summaries of pain and opioid consumption. All three non-randomized case–control studies were found to be of good to high quality (score of 7–9) [31, 39, 53].

### Procedure cost and operating time

**Fig. 2:**
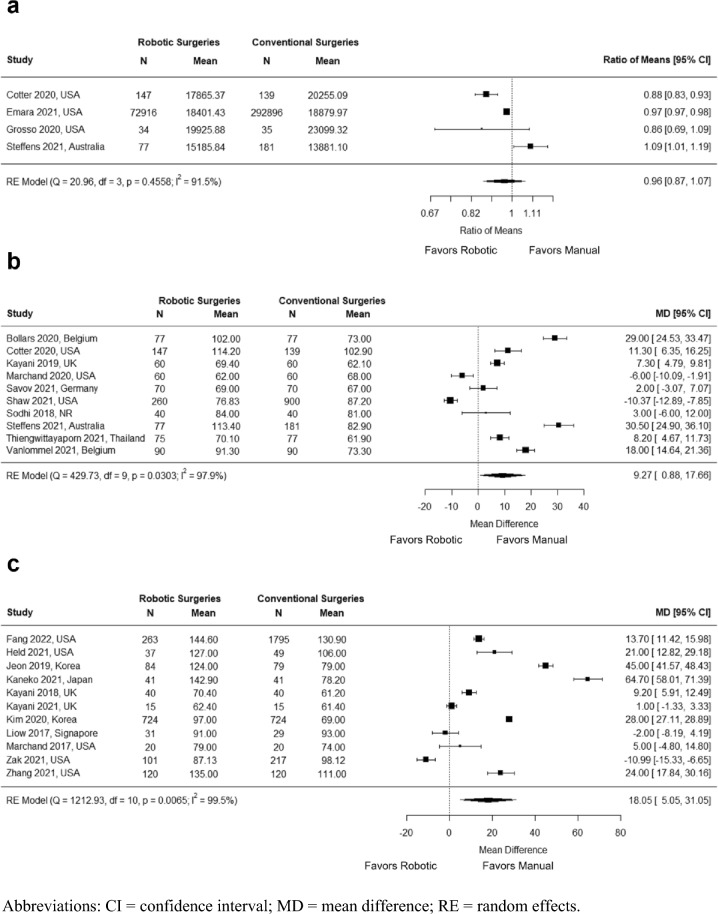
Meta-analyses of **a** Procedure cost **b** Operating time defined as incision to closure **c** General operating time

Total procedure cost (defined as episode cost) was evaluated in four studies with a total of 366,425 knees (366,410 patients). No significant difference in cost between robotic and manual TKA procedures was observed, though numerically there was a 4% reduction in cost for robotic TKA (RoM = 0.96; 95% CI = 0.87, 1.07; *P* = 0.456; *I*^*2*^ = 91.5%) (Fig. 2a).

Operating time was defined in two different ways among the 21 studies that reported this outcome: 10 studies with a total of 2650 knees (2635 patients) reported operating time defined as from incision to closure and 11 studies with a total of 4605 knees (4474 patients) reported the outcome with a general definition (i.e., operating time, surgical time, operation time, etc.). To maximize comparability between studies, the two definitions were analyzed separately. A significantly longer operating time was observed for robotic TKA compared with manual TKA in both analyses (incision to closure definition: WMD = 9.27 min; 95% CI = 0.88, 17.66; *P* = 0.030; *I*^*2*^ = 97.9%; general definition: WMD = 18.05 min; 95% CI = 5.05, 31.05; *P* = 0.006;* I*^*2*^ = 99.5%) (Fig. 2b, Fig. 2c).

### Hospital length of stay and percentage of patients discharged to home

**Fig. 3:**
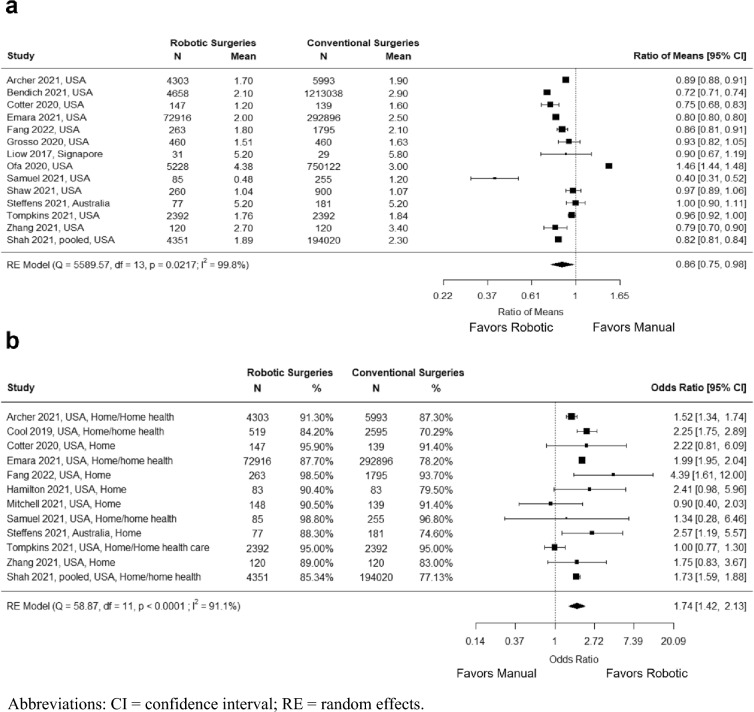
Meta-analyses for **a** Length of stay **b** Percentage of patients discharged to home

Fourteen studies with a total of 2,557,631 knees (2,557,616 patients) reported hospital LOS. A significantly shorter LOS was observed for robotic TKA (RoM = 0.86; 95% CI = 0.75, 0.98; *P* = 0.022; *I*^*2*^ = 99.8%) (Fig. 3a). There were a total of 12 studies with 586,012 knees (585,982 patients) that reported the percentage of patients who were discharged to home. A significantly greater odds of being discharged to home was observed for robotic TKA compared with manual TKA (OR = 1.74; 95% CI = 1.42, 2.13; *P* < 0.001;* I*^*2*^ = 91.1%) (Fig. 3b).

### 90-day readmissions and emergency room visits

**Fig. 4:**
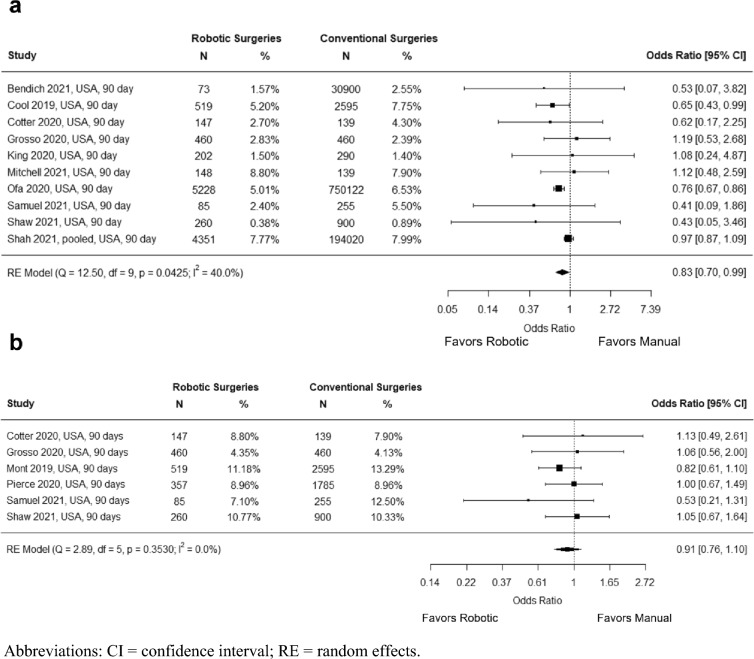
Meta-analyses for **a** 90-day readmission rates **b** 90-Day emergency room visits

Regarding 90-day outcomes after robotic and manual TKA, ten studies with a total of 991,293 knees (991,263 patients) reported the percentage of patients who were readmitted and six studies with a total of 7962 knees (7947 patients) reported the percentage of patients with an ER visit. A significantly lower odds of 90-day readmission was observed for robotic vs. manual TKA (OR = 0.83; 95% CI = 0.70, 0.99; *P* = 0.043;* I*^*2*^ = 40.0%) (Fig. 4a). No difference in 90-day ER visits was observed between robotic and manual TKA (OR = 0.91; 95% CI = 0.76, 1.10; *P* = 0.353; *I*^*2*^ = 0.0%) (Fig. 4b).

### Pain and opioid consumption

Post-operative pain scores were reported in 16 studies [36, 37, 40, 44, 46, 52, 61, 63–71] with more than 6000 patients at various time points using different patient-reported outcome instruments, including the visual analog scale (VAS), the Western Ontario and McMaster Universities Osteoarthritis Index (WOMAC), the Knee Injury and Osteoarthritis Outcome Score (KOOS), the Knee Society Score (KSS), the Numerical Rating Scale (NRS), and a max pain score survey (in which patients were asked to rate their pain on a scale of 1 to 10). Due to the limited data for each instrument and time point, no meta-analyses were conducted and results were narratively summarized.

In the immediate post-operative period (post-operative days 0 to 3), one study found significantly reduced pain scores for robotic TKA compared with manual TKA (*P* < 0.001) [40]. However, similar pain scores for both types of procedures were reported in several other studies [36, 44, 52, 61, 65]. Conclusions from comparative pain outcomes in the short- to long-term post-operative period varied. Two studies reported significantly reduced pain scores for robotic TKA at 6 weeks [66] and 6 months [46], however, other studies found no significant differences in pain scores at similar short- to medium-term follow-up time points [37, 63, 68]. One study found that improvement in pain with walking at 4–6 weeks and 3 months was greater for robotic TKA than with manual TKA, but there was no difference in pain with stairs between groups at the same time points [71]. Studies that reported long-term follow-up (ranging from 1 year to > 10 years) described no significant differences in pain scores between robotic and manual TKA at the final follow-up [37, 63–65, 67–70].

Nine studies with more than 750,000 patients described post-operative opioid consumption for robotic and manual TKA [33, 40, 44, 48, 50, 52, 61, 66, 72]. Again, studies reported a variety of different outcomes at different time points and therefore a meta-analysis was not conducted. Four studies reported robotic TKA was associated with significantly reduced inpatient opiate analgesia and morphine milligram equivalents (MME) compared with manual TKA [40, 48, 52, 72]. Five studies observed outpatient MME for robotic TKA patients was either similar to or less than that of manual TKA patients [33, 50, 61, 66, 72]. One study observed significantly lower total opioid consumption for robotic vs. manual TKA at 90 days, 6 months, and 1 year [50], and another study reported total prescribed opioids at 90 days were reduced by 57% for robotic TKA compared with manual TKA [33]. A third study reported significantly less opioids prescribed to robotic TKA patients than manual TKA patients at discharge, but no difference in total MME prescriptions at three months and beyond [72]. Two additional studies reported no difference in daily MME at 2 weeks between robotic and manual TKA [61, 66], with one of the studies having observed a significantly lower daily opioid use in the robotic group at a longer follow-up of 6 weeks [66] and the other having observed no difference between the groups at up to three-month follow-up [61].

## Discussion

Interest in robotic TKA procedures for osteoarthritis is increasing as evidence on the benefits relative to manual procedures continues to grow. The advantages of robotic TKA compared with manual TKA for clinical outcomes (e.g., alignment, implant precision, etc.) have been well documented [10, 13–22]. Here, we sought to fill an evidence gap by conducting a systematic review and meta-analysis of economic and HRU outcomes for robotic compared with manual TKA.

Our primary findings suggest that there are several HRU outcomes that may favor robotic TKA over manual TKA. We observed a significantly shorter hospital LOS (~ 9.5 h; *P* = 0.022) for the robotic TKA group compared with the manual TKA group. Two previous SLRs have also narratively reported reduced LOS for robotic vs manual TKA [14, 19]. Additionally, compared with manual TKA patients, we found robotic TKA patients had significantly greater odds of being discharged to home (74% greater likelihood;* P* < 0.001). Discharge to home is economically favorable for the overall healthcare system as home-based discharge is associated with lower costs compared to discharge to other inpatient rehabilitation such as skilled nursing facilities [73]. Results of one study with 234 participants randomized to either home health or inpatient rehabilitation following total hip or knee replacement also found home health to be more cost-effective with no difference in functional outcomes [74]. Our study also found robotic TKA patients had significantly lower odds of 90-day readmissions (17% lower likelihood; *P* = 0.043). Further, there were no differences between the two groups for 90-day ER visits or procedure cost. The observation of no difference in procedure cost contrasts with previous SLRs that narratively described significantly lower 30- to 90-day episode of care costs for robotic vs. manual TKA, as well as significantly higher intraoperative costs but significantly lower inpatient costs [14, 19]. The procedure cost outcome was included as it is relevant to clinical and non-clinical stakeholders and hospital decision makers, however this analysis is associated with notable limitations. Firstly, one of the four included studies was not from the US, and given vastly different healthcare costs between countries, this adds significant variability to our analysis. Secondly, the reported costs were not all from the same cost year, adding additional heterogeneity. Lastly, one study had a substantially larger sample size (*N* = 365,812) compared to the others (range: *N* = 69 to 286). Although steps were taken to address these limitations, the results of the analysis should be interpreted with caution.

Operating time was observed to be significantly longer for robotic vs. manual TKA, which aligned with previous meta-analyses [14, 15]. However, we note that several studies have reported robotic TKA operating time was significantly reduced after a short initial learning phase (range across studies: 6 to 43 procedures) [47, 56, 58, 60, 62, 75, 76]. Furthermore, many studies have reported no significant difference between robotic TKA procedures performed after the learning curve and manual TKA [47, 56, 58, 75, 76]. One study that assessed a long-term learning curve of robotic TKA (> 1 year) reported that over time, robotic procedures continued to improve in efficiency, becoming significantly shorter than manual TKA procedures [47]. As operators become more experienced with robotic TKA, further study will be required to assess whether relative operating time of robotic and manual TKA procedures changes in future.

Regarding secondary objectives, although pain and opioid use outcome time points and definitions reported in included studies were too varied to conduct meaningful analyses, review of the identified studies suggest that post-operative pain and opioid consumption are at least similar for robotic and manual TKA or may slightly favor robotic TKA. One previous SLR has also discussed reduced opioid usage with robotic TKA compared to manual TKA [19].

Our economic and healthcare resource utilization findings, coupled with the established clinical and functional benefits of robotic TKA suggest that the use of robotics in TKA has the potential to impact hospitals’ quality improvement initiatives and financial sustainability. As robotic assisted orthopedic surgery continues to advance and evolve, further research, in the form of randomized controlled trials, should seek to effectively quantify the economic advantage of using robotic TKA.

The strengths of this study include a comprehensive systematic review of the literature and inclusion of several studies for most outcomes analyzed. Additionally, limiting studies to the past five years may have helped limit confounding due to historical clinical practice, though the results should be interpreted with the following limitations. Many of the included studies were retrospective observational cohorts, and the lack of randomized controlled trials is apparent in the high heterogeneity observed for many of the analyses (hence, random effects models were used). Study settings and designs differed, including studies from large multi-institution retrospective database reviews, small single institution prospective cohort studies, and a few randomized controlled trials. This heterogeneity in study settings, and particularly a lack of randomized controlled trials, limits our ability to draw firm conclusions regarding the relative economic benefits of robotic and manual TKA. Additionally, for retrospective studies, we did not explore potential confounders such as different time periods for the groups compared, arising from the inclusion of studies that compared more recent robotic TKA cohorts to historic manual TKA cohorts. Another limitation observed in the operating time analyses and for pain and opioid use outcomes was a lack of standardized reporting of outcomes. Finally, most of the analyses predominantly included studies from the US, with a limited number of studies from other countries, which may limit the generalizability of the results to other regions.

## Conclusions

To our knowledge, this is the first meta-analysis comparing robotic and manual TKA for hospital LOS, percentage of patients discharged to home, 90-day readmission, and 90-day ER visits. We observed a significantly shorter hospital LOS, a significantly greater odds of discharge to home, and a significantly lower odds of 90-day readmissions for robotic TKA compared with manual TKA. No differences were found between the groups for 90-day ER visits and procedure cost. Consistent with previous SLRs, we found a significantly longer operating time for robotic procedures compared with manual procedures, and evidence of a robotic TKA learning curve that influences this outcome. A review of pain and opioid use found similar outcomes for robotic and manual TKA. Due to a lack of sufficient evidence in the form of randomized controlled trials, further research is needed to effectively quantify the relative benefits of robotic TKA compared with manual TKA.

### Supplementary Information

Below is the link to the electronic supplementary material.

Supplementary file1 (DOCX 219 KB)

## Data Availability

All data supporting this study are included within the article, its supplementary materials, or cited articles.
