# Supplementary material for: Systematic review and meta-analysis of economic and healthcare resource utilization outcomes for robotic versus manual total knee arthroplasty
Source: J Robot Surg. 2023 Oct 11;17(6):2899–910. doi: 10.1007/s11701-023-01703-x (PMC10678833; doi:10.1007/s11701-023-01703-x)
Supplement: Supplementary file 1 — Supplementary file1 (DOCX 219 KB) [file 11701_2023_1703_MOESM1_ESM.docx]

**Supplemental Material for:**

Systematic Review and Meta-Analysis of Economic and Healthcare Resource Utilization Outcomes for Robotic versus Manual Total Knee Arthroplasty

Daniel Hoeffel, Laura Goldstein*, Dhara Intwala, Lisa Kaindl, Aidan Dineen, Leena Patel, Robert Mayle

*DePuy Synthes, Raynham, Massachusetts, United States, [lgoldste@ITS.JNJ.com](mailto:lgoldste@ITS.JNJ.com)

# Initial Search Strategy

Appendix Table 1: Full search strategy for the initial SLR

| **#** | | **Searches** | **Results** | |
| --- | --- | --- | --- | --- |
| **Embase 1974 to 2019 September 26: accessed September 28th 2019** | | | | |
| 1 | | ((knee* or hip*) adj2 (replacement* or arthroplast*)).mp. | 89936 | |
| 2 | | exp knee arthroplasty/ | 26374 | |
| 3 | | exp hip arthroplasty/ | 22369 | |
| 4 | | (TKA or TKR or UKA or PKA or THA or THR).mp. | 87099 | |
| 5 | | 1 or 2 or 3 or 4 | 152356 | |
| 6 | | (MAKO* or Navio* or OMNIBotic* or ROSA* or Robodoc*).mp. | 22596 | |
| 7 | | (Da Vinci* or Intuitive*).mp. | 25791 | |
| 8 | | (Brainlab* or Knee3*).mp. | 2212 | |
| 9 | | (Robot* adj2 assist*).mp. | 29803 | |
| 10 | | 9 and 5 | 347 | |
| 11 | | joint replacement surgery.mp. | 1895 | |
| 12 | | ((robot* or assist*) adj4 (joint replacement* or joint arthroplast* or hip replacement* or hip arthroplast* or knee replacement* or knee arthroplast*)).mp. | 757 | |
| 13 | | ((Conventional or conventional total or conventional partial or conventional unicompartment* or traditional or manual) adj2 (knee arthroplast* or knee replacement*)).mp. | 240 | |
| 14 | | ((Conventional or conventional total or traditional or manual) adj2 (hip arthroplast* or hip replacement*)).mp. | 344 | |
| 15 | | ((assist* or CAS) adj4 (joint arthroplast* or joint replacement* or joint navigation or knee arthroplast* or knee replacement* or knee navigation)).mp. | 554 | |
| 16 | | ((assist* or CAS) adj4 (hip arthroplast* or hip replacement* or hip navigation)).mp. | 139 | |
| 17 | | exp robotic surgical procedure/ | 2840 | |
| 18 | | exp computer assisted surgery/ | 11953 | |
| 19 | | 6 or 7 or 8 or 10 or 11 or 12 or 13 or 14 or 15 or 16 or 17 or 18 | 64183 | |
| 20 | | (incremental cost effective* ratio or incremental cost utility ratio or ICER or ICUR or Quality Adjusted Life Year* or QALY or economic evaluation or Markov Model* or discrete choice experiment* or resource utilization).mp. | 77235 | |
| 21 | | ((Cost* or economic or cost-effective* or cost-utility or cost-benefit or cost-minimization or budget impact or multi criteria decision) adj2 analy*s).mp. | 240740 | |
| 22 | | (opportunity cost or productivity).mp. | 84151 | |
| 23 | | exp absenteeism/ | 16638 | |
| 24 | | absent*.mp. | 206009 | |
| 25 | | "health care cost"/ or "cost benefit analysis"/ | 251581 | |
| 26 | | economic model/ | 1778 | |
| 27 | | exp return to work/ or exp work resumption/ | 9008 | |
| 28 | | (societ* adj2 cost*).mp. | 4864 | |
| 29 | | (fiscal or financial or finance or funding).tw. | 182111 | |
| 30 | | satisfact*.mp. | 435145 | |
| 31 | | 20 or 21 or 22 or 23 or 24 or 25 or 26 or 27 or 28 or 29 or 30 | 1260237 | |
| 32 | | (Patient reported outcome* or PROM or Oxford Knee Score or OKS or Harris Hip Score or HSS or Visual Analog Scale or VAS or Knee Society Score or KSS or American Knee Society Score or AKSS or WOMAC or "Knee Injury and Osteoarthritis Score" or KOOS or hip knee ankle or UCLA Activity Score or International Knee Society Score or IKS or patient satisfaction or Forgotten Joint Score or "Hip Disability and Osteoarthritis Outcome Score" or HOOS or SF-12 or SF-6 or PROMIS or new knee society score or NKSS or knee society function score or KSF or International Knee Documentation Committee Scale or IKDC or anterior knee pain scale or AKPS or patient's knee implant or PKIP).mp. | 316308 | |
| 33 | | ((qol or hrqol or quality of life).ti,kw. or "quality of life"/) and ((qol or hrqol$ or quality of life) adj2 (increas$ or decrease$ or improv$ or declin$ or reduc$ or high$ or low$ or effect or effects or worse or score or scores or change$1 or impact$1 or impacted or deteriorat$)).ab. | 122462 | |
| 34 | | (eq-5d or eq5d or eq-5 or eq5 or euro qual or euroqual or euro qual5d or euroqual5d or euro qol or euroqol or euro qol5d or euroqol5d or euro quol or euroquol or euro quol5d or euroquol5d or eur qol or eurqol or eur qol5d or eur qol5d or eur?qul or eur?qul5d or euro$ quality of life or european qol).ti,ab,kw. | 19461 | |
| 35 | | (euro$ adj3 (5 d or 5d or 5 dimension$ or 5dimension$ or 5 domain$ or 5domain$)).ti,ab,kw. | 5717 | |
| 36 | | (utility adj3 (score$1 or valu$ or health$ or cost$ or measur$ or disease$ or mean or gain or gains or index$)).ti,ab,kw. | 22879 | |
| 37 | | utilities.ti,ab,kw. | 10991 | |
| 38 | | (sf36$ or sf 36$ or sf thirtysix or sf thirty six).ti,ab,kw. | 36599 | |
| 39 | | (length of stay or LOS).mp. | 249208 | |
| 40 | | readmission*.mp. | 66183 | |
| 41 | | (readmission adj rate*).mp. | 14871 | |
| 42 | | revision*.mp. | 120230 | |
| 43 | | (revision adj rate*).mp. | 3820 | |
| 44 | | (reoperation* or (reoperation adj rate*)).mp. | 95832 | |
| 45 | | (implant adj survival).mp. | 3039 | |
| 46 | | (failure adj rate).mp. | 17276 | |
| 47 | | (operation adj time).mp. | 21068 | |
| 48 | | exp "length of stay"/ | 173582 | |
| 49 | | "procedure time".mp. | 11904 | |
| 50 | | (operating room adj2 (time or footprint)).mp. | 1949 | |
| 51 | | transport*.mp. | 936101 | |
| 52 | | ("theat* time" or "registration time" or "complication* rate*" or "adverse effect*" or "adverse event*").mp. | 566538 | |
| 53 | | ('OR' adj time).mp. | 12345 | |
| 54 | | ((limb or axial or sagittal or femoral or tibial or mechanical or rotational or translational or varus or valgus or anatomic or component) adj3 alignment).mp. | 7367 | |
| 55 | | 32 or 33 or 34 or 35 or 36 or 37 or 38 or 39 or 40 or 41 or 42 or 43 or 44 or 45 or 46 or 47 or 48 or 49 or 50 or 51 or 52 or 53 or 54 | 2395372 | |
| 56 | | 31 or 55 | 3369646 | |
| 57 | | 5 and 19 and 56 | 1662 | |
| 58 | | limit 57 to yr="2000 -Current" | 1596 | |
| **Ovid MEDLINE(R) and Epub Ahead of Print, In-Process & Other Non-Indexed Citations, Daily and Versions(R) 1946 to September 26, 2019: accessed 28th September 2019** | | | | |
| 1 | | ((knee* or hip*) adj2 (replacement* or arthroplast*)).mp. | 67318 | |
| 2 | | exp Arthroplasty, Replacement, Knee/ | 22150 | |
| 3 | | exp Arthroplasty, Replacement, Hip/ | 25383 | |
| 4 | | (TKA or TKR or UKA or PKA or THA or THR).mp. | 71174 | |
| 5 | | 1 or 2 or 3 or 4 | 118113 | |
| 6 | | (MAKO* or Navio* or OMNIBotic* or ROSA* or Robodoc*).mp. | 20743 | |
| 7 | | (Da Vinci* or Intuitive*).mp. | 19168 | |
| 8 | | (Brainlab* or Knee3*).mp. | 535 | |
| 9 | | (Robot* adj2 assist*).mp. | 13141 | |
| 10 | | 5 and 9 | 247 | |
| 11 | | joint replacement surgery.mp. | 1182 | |
| 12 | | ((robot* or assist*) adj4 (joint replacement* or joint arthroplast* or hip replacement* or hip arthroplast* or knee replacement* or knee arthroplast*)).mp. | 668 | |
| 13 | | ((Conventional or conventional total or conventional partial or conventional unicompartment* or traditional or manual) adj2 (knee arthroplast* or knee replacement*)).mp. | 203 | |
| 14 | | ((Conventional or conventional total or traditional or manual) adj2 (hip arthroplast* or hip replacement*)).mp. | 266 | |
| 15 | | ((assist* or CAS) adj4 (joint arthroplast* or joint replacement* or joint navigation or knee arthroplast* or knee replacement* or knee navigation)).mp. | 490 | |
| 16 | | ((assist* or CAS) adj4 (hip arthroplast* or hip replacement* or hip navigation)).mp. | 124 | |
| 17 | | exp Robotic Surgical Procedures/ | 6356 | |
| 18 | | exp Surgery, Computer-Assisted/ | 22393 | |
| 19 | | 6 or 7 or 8 or 10 or 11 or 12 or 13 or 14 or 15 or 16 or 17 or 18 | 63294 | |
| 20 | | (incremental cost effective* ratio or incremental cost utility ratio or ICER or ICUR or Quality Adjusted Life Year* or QALY or economic evaluation or Markov Model* or discrete choice experiment* or resource utilization).mp. | 42404 | |
| 21 | | ((Cost* or economic or cost-effective* or cost-utility or cost-benefit or cost-minimization or budget impact or multi criteria decision) adj2 analy*s).mp. | 139624 | |
| 22 | | (opportunity cost or productivity).mp. | 55396 | |
| 23 | | exp Absenteeism/ | 8818 | |
| 24 | | absent*.mp. | 157778 | |
| 25 | | Health Care Costs/ | 37660 | |
| 26 | | Cost-Benefit Analysis/ | 77981 | |
| 27 | | Models, Economic/ | 9595 | |
| 28 | | exp Return to Work/ | 2162 | |
| 29 | | (societ* adj2 cost*).mp. | 3476 | |
| 30 | | (fiscal or financial or finance or funding).tw. | 138614 | |
| 31 | | satisfact*.mp. | 324127 | |
| 32 | | 20 or 21 or 22 or 23 or 24 or 25 or 26 or 27 or 28 or 29 or 30 or 31 | 831727 | |
| 33 | | (Patient reported outcome* or PROM or Oxford Knee Score or OKS or Harris Hip Score or HSS or Visual Analog Scale or VAS or Knee Society Score or KSS or American Knee Society Score or AKSS or WOMAC or "Knee Injury and Osteoarthritis Score" or KOOS or hip knee ankle or UCLA Activity Score or International Knee Society Score or IKS or patient satisfaction or Forgotten Joint Score or "Hip Disability and Osteoarthritis Outcome Score" or HOOS or SF-12 or SF-6 or PROMIS or new knee society score or NKSS or knee society function score or KSF or International Knee Documentation Committee Scale or IKDC or anterior knee pain scale or AKPS or patient's knee implant or PKIP).mp. | 185922 | |
| 34 | | ((qol or hrqol or quality of life).ti,kw. or "quality of life"/) and ((qol or hrqol$ or quality of life) adj2 (increas$ or decrease$ or improv$ or declin$ or reduc$ or high$ or low$ or effect or effects or worse or score or scores or change$1 or impact$1 or impacted or deteriorat$)).ab. | 51858 | |
| 35 | | (eq-5d or eq5d or eq-5 or eq5 or euro qual or euroqual or euro qual5d or euroqual5d or euro qol or euroqol or euro qol5d or euroqol5d or euro quol or euroquol or euro quol5d or euroquol5d or eur qol or eurqol or eur qol5d or eur qol5d or eur?qul or eur?qul5d or euro$ quality of life or european qol).ti,ab. | 10323 | |
| 36 | | (euro$ adj3 (5 d or 5d or 5 dimension$ or 5dimension$ or 5 domain$ or 5domain$)).ti,ab. | 3602 | |
| 37 | | (utility adj3 (score$1 or valu$ or health$ or cost$ or measur$ or disease$ or mean or gain or gains or index$)).ti,ab. | 13987 | |
| 38 | | utilities.ti,ab. | 6650 | |
| 39 | | (sf36$ or sf 36$ or sf thirtysix or sf thirty six).ti,ab. | 20897 | |
| 40 | | (length of stay or LOS).mp. | 173463 | |
| 41 | | readmission*.mp. | 30646 | |
| 42 | | revision*.mp. | 89871 | |
| 43 | | (reoperation* or re-operation*).mp. [mp=title, abstract, original title, name of substance word, subject heading word, floating sub-heading word, keyword heading word, organism supplementary concept word, protocol supplementary concept word, rare disease supplementary concept word, unique identifier, synonyms] | 105363 | |
| 44 | | (implant adj survival).mp. | 2889 | |
| 45 | | (failure adj rate).mp. | 12368 | |
| 46 | | (operation adj time).mp. | 12520 | |
| 47 | | exp "Length of Stay"/ | 83449 | |
| 48 | | "procedure time".mp. | 5164 | |
| 49 | | (operating room adj2 (time or footprint)).mp. | 1353 | |
| 50 | | transport*.mp. | 727035 | |
| 51 | | ("theat* time" or "registration time" or "complication* rate*" or "adverse effect*" or "adverse event*").mp. | 1891307 | |
| 52 | | ('OR' adj (time or footprint)).mp. | 8276 | |
| 53 | | ((limb or axial or sagittal or femoral or tibial or mechanical or rotational or translational or varus or valgus or anatomic or component) adj3 alignment).mp. | 5771 | |
| 54 | | 33 or 34 or 35 or 36 or 37 or 38 or 39 or 40 or 41 or 42 or 43 or 44 or 45 or 46 or 47 or 48 or 49 or 50 or 51 or 52 or 53 | 3119084 | |
| 55 | | 32 or 54 | 3746459 | |
| 56 | | 5 and 19 and 55 | 1663 | |
| 57 | | limit 56 to yr="2000 -Current" | 1585 | |
| **EBM Reviews - Cochrane Database of Systematic Reviews 2005 to September 11, 2019, EBM Reviews - ACP Journal Club 1991 to August 2019, EBM Reviews - Database of Abstracts of Reviews of Effects 1st Quarter 2016, EBM Reviews - Cochrane Clinical Answers August 2019, EBM Reviews - Cochrane Central Register of Controlled Trials August 2019, EBM Reviews - Cochrane Methodology Register 3rd Quarter 2012, EBM Reviews - Health Technology Assessment 4th Quarter 2016, EBM Reviews - NHS Economic Evaluation Database 1st Quarter 2016: accessed September 28th 2019** | | | | |
| 1 | | ((knee* or hip*) adj2 (replacement* or arthroplast*)).mp. | | 13208 |
| 2 | | exp Arthroplasty, Replacement, Knee/ | | 2493 |
| 3 | | exp Arthroplasty, Replacement, Hip/ | | 1976 |
| 4 | | (TKA or TKR or UKA or PKA or THA or THR).mp. | | 5141 |
| 5 | | 1 or 2 or 3 or 4 | | 13942 |
| 6 | | (MAKO* or Navio* or OMNIBotic* or ROSA* or Robodoc*).mp. | | 1566 |
| 7 | | (Da Vinci* or Intuitive*).mp. | | 1442 |
| 8 | | (Brainlab* or Knee3*).mp. | | 35 |
| 9 | | (Robot* adj2 assist*).mp. | | 2661 |
| 10 | | 5 and 9 | | 60 |
| 11 | | joint replacement surgery.mp. | | 261 |
| 12 | | ((robot* or assist*) adj4 (joint replacement* or joint arthroplast* or hip replacement* or hip arthroplast* or knee replacement* or knee arthroplast*)).mp. | | 231 |
| 13 | | ((Conventional or conventional total or conventional partial or conventional unicompartment* or traditional or manual) adj2 (knee arthroplast* or knee replacement*)).mp. | | 162 |
| 14 | | ((Conventional or conventional total or traditional or manual) adj2 (hip arthroplast* or hip replacement*)).mp. | | 89 |
| 15 | | ((assist* or CAS) adj4 (joint arthroplast* or joint replacement* or joint navigation or knee arthroplast* or knee replacement* or knee navigation)).mp. | | 191 |
| 16 | | ((assist* or CAS) adj4 (hip arthroplast* or hip replacement* or hip navigation)).mp. | | 35 |
| 17 | | exp Robotic Surgical Procedures/ | | 0 |
| 18 | | exp Surgery, Computer-Assisted/ | | 792 |
| 19 | | 6 or 7 or 8 or 10 or 11 or 12 or 13 or 14 or 15 or 16 or 17 or 18 | | 4350 |
| 20 | | (incremental cost effective* ratio or incremental cost utility ratio or ICER or ICUR or Quality Adjusted Life Year* or QALY or economic evaluation or Markov Model* or discrete choice experiment* or resource utilization).mp. | | 28659 |
| 21 | | ((Cost* or economic or cost-effective* or cost-utility or cost-benefit or cost-minimization or budget impact or multi criteria decision) adj2 analy*s).mp. | | 40155 |
| 22 | | (opportunity cost or productivity).mp. | | 4939 |
| 23 | | exp Absenteeism/ | | 543 |
| 24 | | absent*.mp. | | 7731 |
| 25 | | Health Care Costs/ | | 4872 |
| 26 | | Cost-Benefit Analysis/ | | 18800 |
| 27 | | Models, Economic/ | | 1543 |
| 28 | | exp Return to Work/ | | 192 |
| 29 | | (societ* adj2 cost*).mp. | | 1776 |
| 30 | | (fiscal or financial or finance or funding).tw. | | 43058 |
| 31 | | satisfact*.mp. | | 58725 |
| 32 | | 20 or 21 or 22 or 23 or 24 or 25 or 26 or 27 or 28 or 29 or 30 or 31 | | 137257 |
| 33 | | (Patient reported outcome* or PROM or Oxford Knee Score or OKS or Harris Hip Score or HSS or Visual Analog Scale or VAS or Knee Society Score or KSS or American Knee Society Score or AKSS or WOMAC or "Knee Injury and Osteoarthritis Score" or KOOS or hip knee ankle or UCLA Activity Score or International Knee Society Score or IKS or patient satisfaction or Forgotten Joint Score or "Hip Disability and Osteoarthritis Outcome Score" or HOOS or SF-12 or SF-6 or PROMIS or new knee society score or NKSS or knee society function score or KSF or International Knee Documentation Committee Scale or IKDC or anterior knee pain scale or AKPS or patient's knee implant or PKIP).mp. | | 79746 |
| 34 | | ((qol or hrqol or quality of life).ti,kw. or "quality of life"/) and ((qol or hrqol$ or quality of life) adj2 (increas$ or decrease$ or improv$ or declin$ or reduc$ or high$ or low$ or effect or effects or worse or score or scores or change$1 or impact$1 or impacted or deteriorat$)).ab. | | 20553 |
| 35 | | (eq-5d or eq5d or eq-5 or eq5 or euro qual or euroqual or euro qual5d or euroqual5d or euro qol or euroqol or euro qol5d or euroqol5d or euro quol or euroquol or euro quol5d or euroquol5d or eur qol or eurqol or eur qol5d or eur qol5d or eur?qul or eur?qul5d or euro$ quality of life or european qol).ti,ab. | | 7999 |
| 36 | | (euro$ adj3 (5 d or 5d or 5 dimension$ or 5dimension$ or 5 domain$ or 5domain$)).ti,ab. | | 2677 |
| 37 | | (utility adj3 (score$1 or valu$ or health$ or cost$ or measur$ or disease$ or mean or gain or gains or index$)).ti,ab. | | 4437 |
| 38 | | utilities.ti,ab. | | 1053 |
| 39 | | (sf36$ or sf 36$ or sf thirtysix or sf thirty six).ti,ab. | | 10261 |
| 40 | | (length of stay or LOS).mp. | | 26665 |
| 41 | | readmission*.mp. | | 7108 |
| 42 | | revision*.mp. | | 6404 |
| 43 | | (reoperation* or re-operation*).mp. | | 6160 |
| 44 | | (implant adj survival).mp. | | 436 |
| 45 | | (failure adj rate).mp. | | 3019 |
| 46 | | (operation adj time).mp. | | 3420 |
| 47 | | exp "Length of Stay"/ | | 8063 |
| 48 | | "procedure time".mp. | | 2296 |
| 49 | | (operating room adj2 (time or footprint)).mp. | | 457 |
| 50 | | transport*.mp. | | 12079 |
| 51 | | ("theat* time" or "registration time" or "complication* rate*" or "adverse effect*" or "adverse event*").mp. | | 152528 |
| 52 | | ('OR' adj (time or footprint)).mp. | | 368413 |
| 53 | | ((limb or axial or sagittal or femoral or tibial or mechanical or rotational or translational or varus or valgus or anatomic or component) adj3 alignment).mp. | | 638 |
| 54 | | 33 or 34 or 35 or 36 or 37 or 38 or 39 or 40 or 41 or 42 or 43 or 44 or 45 or 46 or 47 or 48 or 49 or 50 or 51 or 52 or 53 | | 566623 |
| 55 | | 32 or 54 | | 622005 |
| 56 | | 5 and 19 and 55 | | 537 |
| 57 | | limit 56 to yr="2000 -Current" [Limit not valid in DARE; records were retained] | | 519 |
| **Econlit 1886 to September 12, 2019: accessed September 28th 2019** | | | | |
| 1 | ((knee* or hip*) adj2 (replacement* or arthroplast*)).mp. | | | 54 |

# Update Search Full Search Strategy

Appendix Table 2: Full search strategy for the update SLR

| **#** | **Searches** | **Results** |
| --- | --- | --- |
| 1 | (knee* adj2 (replacement* or arthroplast*)).mp. | 106774 |
| 2 | exp Arthroplasty, Replacement, Knee/ | 50056 |
| 3 | (TKA or TKR or UKA or PKA).mp. | 107892 |
| 4 | 1 or 2 or 3 | 174899 |
| 5 | (MAKO* or Navio* or OMNIBotic* or ROSA* or Robodoc*).mp. | 53340 |
| 6 | (Da Vinci* or TSolution*).mp. | 12636 |
| 7 | (Brainlab* or Knee3*).mp. | 3421 |
| 8 | (Robot* adj2 assist*).mp. | 64366 |
| 9 | 4 and 8 | 991 |
| 10 | joint replacement surgery.mp. | 3874 |
| 11 | ((robot* or assist*) adj4 (joint replacement* or joint arthroplast* or knee replacement* or knee arthroplast*)).mp. | 1889 |
| 12 | ((Conventional or conventional total or conventional partial or conventional unicompartment* or traditional or manual) adj2 (knee arthroplast* or knee replacement*)).mp. | 787 |
| 13 | ((assist* or CAS) adj4 (joint arthroplast* or joint replacement* or joint navigation or knee arthroplast* or knee replacement* or knee navigation)).mp. | 1716 |
| 14 | exp Surgery, Computer-Assisted/ | 59975 |
| 15 | exp Robotic Surgical Procedures/ | 27949 |
| 16 | 5 or 6 or 7 or 9 or 10 or 11 or 12 or 13 or 14 or 15 | 129875 |
| 17 | (incremental cost effective* ratio or incremental cost utility ratio or ICER or ICUR or Quality Adjusted Life Year* or QALY or economic evaluation or Markov Model* or discrete choice experiment* or resource utilization).mp. | 161318 |
| 18 | ((Cost* or economic or cost-effective* or cost-utility or cost-benefit or cost-minimization or budget impact or multi criteria decision) adj2 analy*s).mp. | 452546 |
| 19 | (opportunity cost or productivity).mp. | 175544 |
| 20 | exp Absenteeism/ | 28485 |
| 21 | absent*.mp. | 411364 |
| 22 | Health Care Costs/ | 214521 |
| 23 | Cost-Benefit Analysis/ | 184633 |
| 24 | Models, Economic/ | 13769 |
| 25 | exp Return to Work/ | 11329 |
| 26 | (societ* adj2 cost*).mp. | 11833 |
| 27 | (fiscal or financial or finance or funding).tw. | 444051 |
| 28 | satisfact*.mp. | 956577 |
| 29 | 17 or 18 or 19 or 20 or 21 or 22 or 23 or 24 or 25 or 26 or 27 or 28 | 2556033 |
| 30 | (Patient reported outcome* or PROM or Oxford Knee Score or OKS or Harris Hip Score or HSS or Visual Analog Scale or VAS or Knee Society Score or KSS or American Knee Society Score or AKSS or WOMAC or "Knee Injury and Osteoarthritis Score" or KOOS or hip knee ankle or UCLA Activity Score or International Knee Society Score or IKS or patient satisfaction or Forgotten Joint Score or "Hip Disability and Osteoarthritis Outcome Score" or HOOS or SF-12 or SF-6 or PROMIS or new knee society score or NKSS or knee society function score or KSF or International Knee Documentation Committee Scale or IKDC or anterior knee pain scale or AKPS or patient's knee implant or PKIP).mp. | 721025 |
| 31 | ((qol or hrqol or quality of life).ti,kw. or "quality of life"/) and ((qol or hrqol$ or quality of life) adj2 (increas$ or decrease$ or improv$ or declin$ or reduc$ or high$ or low$ or effect or effects or worse or score or scores or change$1 or impact$1 or impacted or deteriorat$)).ab. | 248624 |
| 32 | (eq-5d or eq5d or eq-5 or eq5 or euro qual or euroqual or euro qual5d or euroqual5d or euro qol or euroqol or euro qol5d or euroqol5d or euro quol or euroquol or euro quol5d or euroquol5d or eur qol or eurqol or eur qol5d or eur qol5d or eur?qul or eur?qul5d or euro$ quality of life or european qol).ti,ab. | 51326 |
| 33 | (euro$ adj3 (5 d or 5d or 5 dimension$ or 5dimension$ or 5 domain$ or 5domain$)).ti,ab. | 16214 |
| 34 | (utility adj3 (score$1 or valu$ or health$ or cost$ or measur$ or disease$ or mean or gain or gains or index$)).ti,ab. | 49535 |
| 35 | utilities.ti,ab. | 22619 |
| 36 | (sf36$ or sf 36$ or sf thirtysix or sf thirty six).ti,ab. | 78713 |
| 37 | (length of stay or LOS).mp. | 553265 |
| 38 | readmission*.mp. | 140605 |
| 39 | revision*.mp. | 259403 |
| 40 | (reoperation* or re-operation*).mp. | 238795 |
| 41 | (implant adj survival).mp. | 7989 |
| 42 | (failure adj rate).mp. | 37598 |
| 43 | (operation adj time).mp. | 47694 |
| 44 | exp "Length of Stay"/ | 329814 |
| 45 | "procedure time".mp. | 24446 |
| 46 | (operating room adj2 (time or footprint)).mp. | 4390 |
| 47 | transport*.mp. | 1898356 |
| 48 | ("theat* time" or "registration time" or "complication* rate*" or "adverse effect*" or "adverse event*").mp. | 3037701 |
| 49 | ('OR' adj (time or footprint)).mp. | 476797 |
| 50 | ((limb or axial or sagittal or femoral or tibial or mechanical or rotational or translational or varus or valgus or anatomic or component) adj3 alignment).mp. | 17051 |
| 51 | 30 or 31 or 32 or 33 or 34 or 35 or 36 or 37 or 38 or 39 or 40 or 41 or 42 or 43 or 44 or 45 or 46 or 47 or 48 or 49 or 50 | 7115629 |
| 52 | 29 or 51 | 9001953 |
| 53 | 4 and 16 and 52 | 3636 |
| 54 | limit 53 to dt="20190801-20221231" [Limit not valid in ACP Journal Club,CCTR,CDSR,CCA,Embase; records were retained] | 2435 |
| 55 | limit 54 to yr="2019 -Current" | 956 |
| 56 | (address or autobiography or bibliography or biography or comment or dictionary or directory or editorial or "expression of concern" or festschrift or historical article or interactive tutorial or lecture or legal case or legislation or news or newspaper article or patient education handout or personal narrative or portrait or video-audio media or webcast or (letter not (letter and randomized controlled trial))).pt. | 4593636 |
| 57 | exp Animals/ not (exp Animals/ and Humans/) [ANIMAL-ONLY REMOVED] | 17095309 |
| 58 | 55 not (56 or 57) [ANIMAL-ONLY STUDIES & OPINION PIECES REMOVED] | 856 |
| 59 | 58 use ppez [MEDLINE results] | 359 |
| 60 | (knee* adj2 (replacement* or arthroplast*)).mp. | 106774 |
| 61 | exp knee arthroplasty/ | 63165 |
| 62 | (TKA or TKR or UKA or PKA).mp. | 107892 |
| 63 | 60 or 61 or 62 | 174899 |
| 64 | (MAKO* or Navio* or OMNIBotic* or ROSA* or Robodoc*).mp. | 53340 |
| 65 | (Da Vinci* or TSolution*).mp. | 12636 |
| 66 | (Brainlab* or Knee3*).mp. | 3421 |
| 67 | (Robot* adj2 assist*).mp. | 64366 |
| 68 | 63 and 67 | 991 |
| 69 | joint replacement surgery.mp. | 3874 |
| 70 | ((robot* or assist*) adj4 (joint replacement* or joint arthroplast* or knee replacement* or knee arthroplast*)).mp. | 1889 |
| 71 | ((Conventional or conventional total or conventional partial or conventional unicompartment* or traditional or manual) adj2 (knee arthroplast* or knee replacement*)).mp. | 787 |
| 72 | ((assist* or CAS) adj4 (joint arthroplast* or joint replacement* or joint navigation or knee arthroplast* or knee replacement* or knee navigation)).mp. | 1716 |
| 73 | exp computer assisted surgery/ | 59975 |
| 74 | exp robotic surgical procedure/ | 27949 |
| 75 | 64 or 65 or 66 or 68 or 69 or 70 or 71 or 72 or 73 or 74 | 129875 |
| 76 | (incremental cost effective* ratio or incremental cost utility ratio or ICER or ICUR or Quality Adjusted Life Year* or QALY or economic evaluation or Markov Model* or discrete choice experiment* or resource utilization).mp. | 161318 |
| 77 | ((Cost* or economic or cost-effective* or cost-utility or cost-benefit or cost-minimization or budget impact or multi criteria decision) adj2 analy*s).mp. | 452546 |
| 78 | (opportunity cost or productivity).mp. | 175544 |
| 79 | exp absenteeism/ | 28485 |
| 80 | absent*.mp. | 411364 |
| 81 | "health care cost"/ | 249779 |
| 82 | "cost benefit analysis"/ | 184633 |
| 83 | economic model/ | 13769 |
| 84 | exp return to work/ or exp work resumption/ | 14815 |
| 85 | (societ* adj2 cost*).mp. | 11833 |
| 86 | (fiscal or financial or finance or funding).tw. | 444051 |
| 87 | satisfact*.mp. | 956577 |
| 88 | 76 or 77 or 78 or 79 or 80 or 81 or 82 or 83 or 84 or 85 or 86 or 87 | 2580531 |
| 89 | (Patient reported outcome* or PROM or Oxford Knee Score or OKS or Harris Hip Score or HSS or Visual Analog Scale or VAS or Knee Society Score or KSS or American Knee Society Score or AKSS or WOMAC or "Knee Injury and Osteoarthritis Score" or KOOS or hip knee ankle or UCLA Activity Score or International Knee Society Score or IKS or patient satisfaction or Forgotten Joint Score or "Hip Disability and Osteoarthritis Outcome Score" or HOOS or SF-12 or SF-6 or PROMIS or new knee society score or NKSS or knee society function score or KSF or International Knee Documentation Committee Scale or IKDC or anterior knee pain scale or AKPS or patient's knee implant or PKIP).mp. | 721025 |
| 90 | ((qol or hrqol or quality of life).ti,kw. or "quality of life"/) and ((qol or hrqol$ or quality of life) adj2 (increas$ or decrease$ or improv$ or declin$ or reduc$ or high$ or low$ or effect or effects or worse or score or scores or change$1 or impact$1 or impacted or deteriorat$)).ab. | 248624 |
| 91 | (eq-5d or eq5d or eq-5 or eq5 or euro qual or euroqual or euro qual5d or euroqual5d or euro qol or euroqol or euro qol5d or euroqol5d or euro quol or euroquol or euro quol5d or euroquol5d or eur qol or eurqol or eur qol5d or eur qol5d or eur?qul or eur?qul5d or euro$ quality of life or european qol).ti,ab,kw. | 51702 |
| 92 | (euro$ adj3 (5 d or 5d or 5 dimension$ or 5dimension$ or 5 domain$ or 5domain$)).ti,ab,kw. | 16256 |
| 93 | (utility adj3 (score$1 or valu$ or health$ or cost$ or measur$ or disease$ or mean or gain or gains or index$)).ti,ab,kw. | 49969 |
| 94 | utilities.ti,ab,kw. | 22783 |
| 95 | (sf36$ or sf 36$ or sf thirtysix or sf thirty six).ti,ab,kw. | 79587 |
| 96 | (length of stay or LOS).mp. | 553265 |
| 97 | readmission*.mp. | 140605 |
| 98 | revision*.mp. | 259403 |
| 99 | (reoperation* or re-operation*).mp. | 238795 |
| 100 | (implant adj survival).mp. | 7989 |
| 101 | (failure adj rate).mp. | 37598 |
| 102 | (operation adj time).mp. | 47694 |
| 103 | exp "length of stay"/ | 329814 |
| 104 | "procedure time".mp. | 24446 |
| 105 | (operating room adj2 (time or footprint)).mp. | 4390 |
| 106 | transport*.mp. | 1898356 |
| 107 | ("theat* time" or "registration time" or "complication* rate*" or "adverse effect*" or "adverse event*").mp. | 3037701 |
| 108 | ('OR' adj (time or footprint)).mp. | 476797 |
| 109 | ((limb or axial or sagittal or femoral or tibial or mechanical or rotational or translational or varus or valgus or anatomic or component) adj3 alignment).mp. | 17051 |
| 110 | 89 or 90 or 91 or 92 or 93 or 94 or 95 or 96 or 97 or 98 or 99 or 100 or 101 or 102 or 103 or 104 or 105 or 106 or 107 or 108 or 109 | 7116472 |
| 111 | 88 or 110 | 9023203 |
| 112 | 63 and 75 and 111 | 3639 |
| 113 | limit 112 to dc="20190801-20221231" [Limit not valid in ACP Journal Club,CCTR,CDSR,CCA; records were retained] | 924 |
| 114 | limit 113 to yr="2019 -Current" | 541 |
| 115 | (exp animal/ or exp animal experimentation/ or exp animal model/ or exp animal experiment/ or nonhuman/ or exp vertebrate/) not (exp human/ or exp human experimentation/ or exp human experiment/) [ANIMAL-ONLY REMOVED] | 11770672 |
| 116 | (comment or editorial or news or newspaper article or (letter not (letter and randomized controlled trial))).pt. [OPINION PIECES REMOVED] | 4157635 |
| 117 | 114 not (115 or 116) [ANIMAL-ONLY STUDIES & OPINION PIECES REMOVED] | 529 |
| 118 | 117 use oemezd [Embase results] | 401 |
| 119 | (knee* adj2 (replacement* or arthroplast*)).mp. | 106774 |
| 120 | exp Arthroplasty, Replacement, Knee/ | 50056 |
| 121 | (TKA or TKR or UKA or PKA).mp. | 107892 |
| 122 | 119 or 120 or 121 | 174899 |
| 123 | (MAKO* or Navio* or OMNIBotic* or ROSA* or Robodoc*).mp. | 53340 |
| 124 | (Da Vinci* or TSolution*).mp. | 12636 |
| 125 | (Brainlab* or Knee3*).mp. | 3421 |
| 126 | (Robot* adj2 assist*).mp. | 64366 |
| 127 | 122 and 126 | 991 |
| 128 | joint replacement surgery.mp. | 3874 |
| 129 | ((robot* or assist*) adj4 (joint replacement* or joint arthroplast* or knee replacement* or knee arthroplast*)).mp. | 1889 |
| 130 | ((Conventional or conventional total or conventional partial or conventional unicompartment* or traditional or manual) adj2 (knee arthroplast* or knee replacement*)).mp. | 787 |
| 131 | ((assist* or CAS) adj4 (joint arthroplast* or joint replacement* or joint navigation or knee arthroplast* or knee replacement* or knee navigation)).mp. | 1716 |
| 132 | exp Surgery, Computer-Assisted/ | 59975 |
| 133 | 123 or 124 or 125 or 127 or 128 or 129 or 130 or 131 or 132 | 129875 |
| 134 | (incremental cost effective* ratio or incremental cost utility ratio or ICER or ICUR or Quality Adjusted Life Year* or QALY or economic evaluation or Markov Model* or discrete choice experiment* or resource utilization).mp. | 161318 |
| 135 | ((Cost* or economic or cost-effective* or cost-utility or cost-benefit or cost-minimization or budget impact or multi criteria decision) adj2 analy*s).mp. | 452546 |
| 136 | (opportunity cost or productivity).mp. | 175544 |
| 137 | exp Absenteeism/ | 28485 |
| 138 | absent*.mp. | 411364 |
| 139 | Health Care Costs/ | 214521 |
| 140 | Cost-Benefit Analysis/ | 184633 |
| 141 | Models, Economic/ | 13769 |
| 142 | exp Return to Work/ | 11329 |
| 143 | (societ* adj2 cost*).mp. | 11833 |
| 144 | (fiscal or financial or finance or funding).tw. | 444051 |
| 145 | satisfact*.mp. | 956577 |
| 146 | 134 or 135 or 136 or 137 or 138 or 139 or 140 or 141 or 142 or 143 or 144 or 145 | 2556033 |
| 147 | (Patient reported outcome* or PROM or Oxford Knee Score or OKS or Harris Hip Score or HSS or Visual Analog Scale or VAS or Knee Society Score or KSS or American Knee Society Score or AKSS or WOMAC or "Knee Injury and Osteoarthritis Score" or KOOS or hip knee ankle or UCLA Activity Score or International Knee Society Score or IKS or patient satisfaction or Forgotten Joint Score or "Hip Disability and Osteoarthritis Outcome Score" or HOOS or SF-12 or SF-6 or PROMIS or new knee society score or NKSS or knee society function score or KSF or International Knee Documentation Committee Scale or IKDC or anterior knee pain scale or AKPS or patient's knee implant or PKIP).mp. | 721025 |
| 148 | ((qol or hrqol or quality of life).ti,kw. or "quality of life"/) and ((qol or hrqol$ or quality of life) adj2 (increas$ or decrease$ or improv$ or declin$ or reduc$ or high$ or low$ or effect or effects or worse or score or scores or change$1 or impact$1 or impacted or deteriorat$)).ab. | 248624 |
| 149 | (eq-5d or eq5d or eq-5 or eq5 or euro qual or euroqual or euro qual5d or euroqual5d or euro qol or euroqol or euro qol5d or euroqol5d or euro quol or euroquol or euro quol5d or euroquol5d or eur qol or eurqol or eur qol5d or eur qol5d or eur?qul or eur?qul5d or euro$ quality of life or european qol).ti,ab. | 51326 |
| 150 | (euro$ adj3 (5 d or 5d or 5 dimension$ or 5dimension$ or 5 domain$ or 5domain$)).ti,ab. | 16214 |
| 151 | (utility adj3 (score$1 or valu$ or health$ or cost$ or measur$ or disease$ or mean or gain or gains or index$)).ti,ab. | 49535 |
| 152 | utilities.ti,ab. | 22619 |
| 153 | (sf36$ or sf 36$ or sf thirtysix or sf thirty six).ti,ab. | 78713 |
| 154 | (length of stay or LOS).mp. | 553265 |
| 155 | readmission*.mp. | 140605 |
| 156 | revision*.mp. | 259403 |
| 157 | (reoperation* or re-operation*).mp. | 238795 |
| 158 | (implant adj survival).mp. | 7989 |
| 159 | (failure adj rate).mp. | 37598 |
| 160 | (operation adj time).mp. | 47694 |
| 161 | exp "Length of Stay"/ | 329814 |
| 162 | "procedure time".mp. | 24446 |
| 163 | (operating room adj2 (time or footprint)).mp. | 4390 |
| 164 | transport*.mp. | 1898356 |
| 165 | ("theat* time" or "registration time" or "complication* rate*" or "adverse effect*" or "adverse event*").mp. | 3037701 |
| 166 | ('OR' adj (time or footprint)).mp. | 476797 |
| 167 | ((limb or axial or sagittal or femoral or tibial or mechanical or rotational or translational or varus or valgus or anatomic or component) adj3 alignment).mp. | 17051 |
| 168 | 147 or 148 or 149 or 150 or 151 or 152 or 153 or 154 or 155 or 156 or 157 or 158 or 159 or 160 or 161 or 162 or 163 or 164 or 165 or 166 or 167 | 7115629 |
| 169 | 146 or 168 | 9001953 |
| 170 | 122 and 133 and 169 | 3636 |
| 171 | 170 and (201908* or 201909* or 201910* or 201911* or 201912* or 2020* or 2021* or 2022*).up. | 2405 |
| 172 | limit 171 to yr="2019 -Current" | 960 |
| 173 | 172 use coch,cctr,cca,acp [EMBR databases results] | 125 |
| 174 | 59 or 118 or 173 | 885 |
| 175 | remove duplicates from 174 | 581 |

# PICOS Criteria

Appendix Table 3: PICOS criteria for the initial SLR

| Criteria | Inclusion criteria | Exclusion criteria |
| --- | --- | --- |
| Population | - Adults with osteoarthritis undergoing TKA, PKA/UKA or THA | - Paediatric patients - Oncology patients - Immunosuppressed patients - Patients requiring replacements due to fracture - Patients undergoing revision arthroplasty |
| Intervention/ comparators | The following robotic systems were of interest:   - MAKO, Navio, OMNIBotics, ROSA - Acrobot Robodoc - Intuitive/da Vinci | - Studies reporting on manual arthroplasty only will be excluded |
| Outcomes | Data of interest included:   - Direct costs (medical, drug costs, staff costs, diagnostic tests, etc.) - Cost-effectiveness estimates (e.g. ICERs, ICURs) - Indirect costs (opportunity cost/productivity loss) - Set-up time - Transportation costs / Storage costs - QALYs - PROMs - Clinician / staff satisfaction (usability of robot) - Resource utilisation - OR time, OR footprint, registration time, procedure time - Readmissions, reoperations and complications, failures, revisions, implant survival) - LOS - Alignment   Studies reporting clinical outcomes only were tagged but were not eligible for extraction. | - Those not listed |
| Study design | No restriction and included the following:  *Economic evaluations*   - Cost-effectiveness analyses - Cost-utility analyses - Budget impact analyses - Cost-minimisation analyses - Cost-benefit analyses - Return on investment   *Clinical/cost studies*   - RCTs - Single arm clinical studies - Comparative/non-comparative observational studies - Case-control studies - Cross-sectional studies - Case series/case reports - Reviews/Editorials/Meta-analyses | - Animal/In vitro studies - Conference abstracts identified via the electronic database search were tagged but not extracted due to limited reporting of patient population, study design, and outcome data |
| Territory of interest | No restriction | |
| Date of publication | Studies published post 2000 | |
| Language of publication | English language publication or non-English language publications with an English abstract | |

Abbreviations: ICER, incremental cost-effectiveness ratio; ICUR, incremental cost utility ratio; LOS, length of stay; OR, operating room; PKA, partial knee arthroplasty; PROM, patient-reported outcome measure; QALY, quality-adjusted life year; RCT, randomised controlled trial; THA, total hip arthroplasty; TKA, total knee arthroplasty; UKA, unicompartmental knee arthroplasty.

Appendix Table 4: PICOS criteria for the updated SLR

| Component | Inclusion criteria | Exclusion criteria |
| --- | --- | --- |
| Population | - Adults with osteoarthritis undergoing robotic TKA, unilateral knee arthroplasty (robotic) | - Pediatric patients - Oncology or immunosuppressed patients - Patients requiring replacements due to fracture - Patients undergoing revision arthroplasty |
| Intervention/ comparator | - MAKO, Navio, OMNIBotics, ROSA, ThinkSolution, Acrobot Robodoc | - Studies reporting on manual arthroplasty only |
| Outcomes | Data of interest included:   - Direct costs (medical, drug, staff, diagnostic tests, etc.) - Cost-effectiveness estimates  (eg, ICERs, ICURs) - Indirect costs (opportunity cost/productivity loss, set-up time, transportation) - QALYs or PROMs - Clinician/staff satisfaction (robot usability) - Resource utilization (operating room time, operating room footprint, registration time, procedure time, readmissions, reoperations and complications, failures, revisions, implant survival, length of stay) | - Those not listed |
| Study design | *Economic evaluations*   - Cost-effectiveness analyses/ Markov models - Cost-utility analyses - Budget impact analyses - Cost-minimization analyses - Cost-benefit analyses - Return on investment   *Cost studies*   - Randomized controlled trials - Single arm clinical studies - Comparative/non-comparative observational studies - Case-control studies - Cross-sectional studies - Case series/case reports - Reviews/Editorials/Meta-analyses | - Animal/In vitro studies - Study protocols - Case study (single case) - Conference abstracts |
| Territory of interest | No restriction | |
| Date of publication | Studies published post 2019 | |
| Language of publication | English language publication or non-English language publications with an English abstract | |

Abbreviations: ICER, incremental cost-effectiveness ratio; ICUR, incremental cost utility ratio; PROM, patient-reported outcome measure; QALY, quality-adjusted life year; TKA, total knee arthroplasty.

# Included Studies

Appendix Table 5: List of studies included in the robotic vs. manual TKA SLR and meta-analysis

| **Study** | **Year** | **Study Design** | **Robot(s) Used** |
| --- | --- | --- | --- |
| Archer [[1](#_ENREF_1)] | 2021 | Retrospective comparative | MAKO |
| Bendich [[2](#_ENREF_2)] | 2021 | Retrospective comparative | NR |
| Bhimani [[3](#_ENREF_3)] | 2020 | Retrospective comparative | MAKO |
| Blum [[4](#_ENREF_4)] | 2021 | Prospective comparative | OMNIBotics |
| Bollars [[5](#_ENREF_5)] | 2020 | Retrospective comparative | NAVIO |
| Cho [[6](#_ENREF_6)] | 2019 | Retrospective comparative | ROBODOC |
| Cool [[7](#_ENREF_7)] | 2019 | Retrospective comparative | MAKO |
| Cotter [[8](#_ENREF_8)] | 2020 | Retrospective comparative | MAKO |
| Emara [[9](#_ENREF_9)] | 2021 | Retrospective comparative | MAKO; ROSA; NAVIO; ROBODOC |
| Fang [[10](#_ENREF_10)] | 2022 | Retrospective comparative | MAKO |
| Greiner [[11](#_ENREF_11)] | 2020 | Retrospective comparative | NR |
| Grosso [[12](#_ENREF_12)] | 2020 | Retrospective comparative | NR |
| Hamilton [[13](#_ENREF_13)] | 2021 | Retrospective comparative | NR |
| Held [[14](#_ENREF_14)] | 2021 | Retrospective comparative | NAVIO |
| Held [[15](#_ENREF_15)] | 2021 | Retrospective comparative | NAVIO |
| Jeon [[16](#_ENREF_16)] | 2019 | Retrospective comparative | ROBODOC |
| Kaneko [[17](#_ENREF_17)] | 2021 | Retrospective comparative | NAVIO |
| Kayani [[18](#_ENREF_18)] | 2018 | Prospective comparative | MAKO |
| Kayani [[19](#_ENREF_19)] | 2019 | Prospective comparative | NR |
| Kayani [[20](#_ENREF_20)] | 2021 | Prospective RCT | MAKO |
| Khlopas [[21](#_ENREF_21)] | 2020 | Prospective comparative | MAKO |
| Kim [[22](#_ENREF_22)] | 2020 | Prospective RCT | ROBODOC |
| King [[23](#_ENREF_23)] | 2020 | Retrospective comparative | MAKO |
| Liow [[24](#_ENREF_24)] | 2017 | Prospective RCT | ROBODOC |
| Mahoney [[25](#_ENREF_25)] | 2020 | Prospective comparative | MAKO |
| Marchand [[26](#_ENREF_26)] | 2017 | Prospective comparative | MAKO |
| Marchand [[27](#_ENREF_27)] | 2019 | Retrospective comparative | NR |
| Marchand [[28](#_ENREF_28)] | 2020 | Retrospective comparative | MAKO |
| Marchand [[29](#_ENREF_29)] | 2021 | Retrospective comparative | MAKO |
| Mitchell [[30](#_ENREF_30)] | 2021 | Retrospective comparative | MAKO |
| Mont [[31](#_ENREF_31)] | 2019 | Retrospective comparative | NR |
| Naziri [[32](#_ENREF_32)] | 2019 | Retrospective comparative | MAKO |
| Ofa [[33](#_ENREF_33)] | 2020 | Retrospective comparative | MAKO |
| Pelkowski [[34](#_ENREF_34)] | 2020 | Retrospective comparative | MAKO |
| Pierce [[35](#_ENREF_35)] | 2020 | Retrospective comparative | NR |
| Samuel [[36](#_ENREF_36)] | 2021 | Retrospective comparative | MAKO |
| Savov [[37](#_ENREF_37)] | 2021 | Retrospective comparative | NAVIO |
| Shah [[38](#_ENREF_38)] | 2021 | Retrospective comparative | NR |
| Shaw [[39](#_ENREF_39)] | 2021 | Prospective comparative | MAKO |
| Smith [[40](#_ENREF_40)] | 2019 | Prospective comparative | MAKO |
| Sodhi [[41](#_ENREF_41)] | 2018 | Retrospective comparative | MAKO |
| Steffens [[42](#_ENREF_42)] | 2021 | Retrospective comparative | MAKO |
| Thiengwittayaporn [[43](#_ENREF_43)] | 2021 | Prospective RCT | NAVIO |
| Tompkins [[44](#_ENREF_44)] | 2021 | Retrospective comparative | MAKO |
| Tompkins [[45](#_ENREF_45)] | 2021 | Retrospective comparative | MAKO |
| Vanlommel [[46](#_ENREF_46)] | 2021 | Retrospective comparative | ROSA |
| Vermue [[47](#_ENREF_47)] | 2020 | Retrospective comparative | MAKO |
| Yang [[48](#_ENREF_48)] | 2017 | Retrospective comparative | ROBODOC |
| Zak [[49](#_ENREF_49)] | 2021 | Retrospective comparative | NAVIO; MAKO |
| Zhang [[50](#_ENREF_50)] | 2021 | Retrospective comparative | MAKO |

Abbreviations: NR = not reported; RCT = randomized controlled trial; SLR = systematic literature review; TKA = total knee arthroplasty.

# Data Extraction

The following study details were extracted into a dedicated data extraction form: study name, year published, country of origin, study design, study time frame, number of patients, treatment arms, key patient baseline characteristics (age, sex, body mass index), follow-up time, indication for surgery, intervention and comparator, type of robot, and brand of implant used. Outcomes extracted included procedure cost, healthcare resource utilization, perioperative outcomes, and patient-reported outcomes.

# Quality Assessment

Appendix Table 6: Results of quality assessment of RCT studies using the NICE Single Technology Appraisal Evidence Submission Checklist

| **Study** | **Year** | **Was randomization carried out appropriately?** | **Was the concealment of treatment allocation adequate?** | **Were the groups similar at the outset of the study in terms of prognostic factors?** | **Were the care providers, participants, and the outcome assessors blind to treatment allocation?** | **Were there any unexpected imbalances in drop-outs between groups?** | **Is there any evidence to suggest that the authors measured more outcomes than they reported?** | **Did the analysis include an intention-to-treat analysis? If so, was this appropriate and were appropriate methods used to account for missing data?** |
| --- | --- | --- | --- | --- | --- | --- | --- | --- |
| Kayani [[20](#_ENREF_20)] | 2021 | Yes | Not clear | Yes | Yes | No | No | Yes |
| Kim [[22](#_ENREF_22)] | 2020 | Yes | Yes | Yes | No | No | No | No |
| Liow [[24](#_ENREF_24)] | 2017 | Yes | No | Not clear | No | No | No | Yes |
| Thiengwitta-yaporn [[43](#_ENREF_43)] | 2021 | Yes | Not clear | Yes | Yes | No | No | Yes |

Abbreviations: RCT = randomized controlled trial; NICE = National Institute for Health and Care Excellence.

Appendix Table 7: Results of quality assessment of non-randomized comparative cohort studies using the Newcastle-Ottawa Non-randomized Cohort Study Tool

|  |  | **Selection** | | | | **Comparability** | **Outcome** | | | **Score^i^** |
| --- | --- | --- | --- | --- | --- | --- | --- | --- | --- | --- |
| **Study** | **Year** | **Representa-tiveness of Exposed Cohort^a^** | **Selection of the Non-Exposed Cohort^b^** | **Ascertainment of Exposure^c^** | **Demonstration that the Outcome of Interest was not Present at the Start of the Study^d^** | **Comparability of the Cohorts on the Basis of Design or Analysis^e^** | **Assessment of Outcome^f^** | **Was the Follow-up Long Enough for Outcomes to Occur?^g^** | **Adequacy of Follow-up of Cohorts^h^** |  |
| Archer [[1](#_ENREF_1)] | 2021 | b | a | a | a | b | b | a | a | 8 |
| Bendich [[2](#_ENREF_2)] | 2021 | b | a | a | a | c | b | a | a | 9 |
| Bhimani [[3](#_ENREF_3)] | 2020 | b | a | a | a | c | b | a | a | 9 |
| Blum [[4](#_ENREF_4)] | 2021 | b | b | a | a | d | c | a | b | 5 |
| Cho [[6](#_ENREF_6)] | 2019 | b | c | a | a | d | c | a | b | 5 |
| Cool [[7](#_ENREF_7)] | 2019 | a | a | a | a | c | b | a | a | 9 |
| Cotter [[8](#_ENREF_8)] | 2020 | b | a | a | a | c | b | a | a | 9 |
| Emara [[9](#_ENREF_9)] | 2021 | a | a | a | a | c | b | b | a | 8 |
| Fang [[10](#_ENREF_10)] | 2022 | b | a | a | a | b | b | a | a | 8 |
| Greiner [[11](#_ENREF_11)] | 2020 | b | a | a | a | c | b | a | a | 9 |
| Grosso [[12](#_ENREF_12)] | 2020 | b | a | a | a | c | b | a | c | 8 |
| Hamilton [[13](#_ENREF_13)] | 2021 | b | a | a | a | c | b | b | a | 8 |
| Held [[14](#_ENREF_14)] | 2021 | b | a | a | a | b | b | a | a | 8 |
| Held [[15](#_ENREF_15)] | 2021 | b | a | a | a | b | b | a | a | 8 |
| Jeon [[16](#_ENREF_16)] | 2019 | b | a | a | a | b | b | a | b | 8 |
| Kayani [[18](#_ENREF_18)] | 2018 | b | a | a | a | c | a | b | a | 8 |
| Kayani [[19](#_ENREF_19)] | 2019 | b | a | a | a | c | a | b | a | 8 |
| Khlopas [[21](#_ENREF_21)] | 2020 | b | a | a | a | b | c | a | a | 7 |
| King [[23](#_ENREF_23)] | 2020 | b | a | a | a | d | b | a | b | 7 |
| Mahoney [[25](#_ENREF_25)] | 2020 | b | a | a | a | c | b | a | b | 9 |
| Marchand [[26](#_ENREF_26)] | 2017 | b | a | a | a | b | c | a | a | 7 |
| Marchand [[27](#_ENREF_27)] | 2019 | b | a | a | a | c | c | a | a | 8 |
| Marchand [[28](#_ENREF_28)] | 2020 | b | a | a | a | b | b | b | a | 7 |
| Marchand [[29](#_ENREF_29)] | 2021 | b | a | a | a | b | b | a | a | 8 |
| Mitchell [[30](#_ENREF_30)] | 2021 | b | a | a | a | b | b | a | c | 7 |
| Mont [[31](#_ENREF_31)] | 2019 | b | a | a | a | c | b | a | c | 8 |
| Naziri [[32](#_ENREF_32)] | 2019 | b | a | a | a | c | b | a | b | 9 |
| Ofa [[33](#_ENREF_33)] | 2020 | a | a | a | a | c | b | a | a | 9 |
| Pelkowski [[34](#_ENREF_34)] | 2020 | b | a | a | b | c | b | a | a | 8 |
| Pierce [[35](#_ENREF_35)] | 2020 | a | a | a | a | c | b | a | a | 9 |
| Samuel [[36](#_ENREF_36)] | 2021 | b | a | a | a | c | b | a | b | 9 |
| Shah [[38](#_ENREF_38)] | 2021 | b | a | a | a | c | b | a | d | 8 |
| Shaw [[39](#_ENREF_39)] | 2021 | b | a | a | a | c | c | a | c | 7 |
| Smith [[40](#_ENREF_40)] | 2019 | b | a | a | a | b | b | a | a | 8 |
| Sodhi [[41](#_ENREF_41)] | 2018 | b | a | a | a | b | b | a | a | 8 |
| Steffens [[42](#_ENREF_42)] | 2021 | b | a | a | a | b | b | a | d | 7 |
| Tompkins [[44](#_ENREF_44)] | 2021 | b | a | a | a | c | b | a | a | 9 |
| Tompkins [[45](#_ENREF_45)] | 2021 | b | a | a | a | c | b | a | a | 9 |
| Vanlommel [[46](#_ENREF_46)] | 2021 | b | a | a | a | c | a | a | a | 9 |
| Vermue [[47](#_ENREF_47)] | 2020 | b | a | a | a | c | a | a | c | 8 |
| Yang [[48](#_ENREF_48)] | 2017 | b | a | a | a | b | a | a | b | 8 |
| Zak [[49](#_ENREF_49)] | 2021 | b | a | a | a | c | b | a | a | 9 |
| Zhang [[50](#_ENREF_50)] | 2021 | b | a | a | a | d | a | a | a | 7 |

^a^ Possible assessments: a) truly representative of the average patient in the community (*); b) somewhat representative of the average patient in the community (*); c) selected group of patients eg nurses, volunteers; d) no description of the derivation of the cohort.

^b^ Possible assessments: a) drawn from the same community as the exposed cohort (*); b) drawn from a different source; c) no description of the derivation of the non exposed cohort.

^c^ Possible assessments: a) secure record (eg, surgical records) (*); b) structured interview (*); c) written self report; d) no description.

^d^ Possible assessments: a) yes (*); b) no.

^e^ Possible assessments: a) study controls for most important factor (*); b) study controls for additional factor (*); c) study controls for most important and additional factor (**); d) no univariate or multivariate analyses or matching.

^f^ Possible assessments: a) independent blind assessment (*); b) record linkage (*); c) self report; d) no description.

^g^ Possible assessments: a) yes (*); b) no.

^h^ Possible assessments: a) complete follow-up - all subjects accounted for (*); b) subjects lost to follow-up unlikely to introduce bias - small number lost - >% follow-up, or description provided of those lost (*); c) follow-up rate <% and no description of those lost; d) no statement.

^i^ Sum of stars.

Appendix Table 8: Results of quality assessment of non-randomized comparative case-control studies using the Newcastle-Ottawa Non-randomized Case-Control Study Tool

|  |  | **Selection** | | | | **Comparability** | **Exposure** | | | **Score^i^** |
| --- | --- | --- | --- | --- | --- | --- | --- | --- | --- | --- |
| **Study** | **Year** | **Is the Case Definition Adequate?^a^** | **Representat-iveness of the Cases^b^** | **Selection of Controls^c^** | **Definition of Controls^d^** | **Comparability of Cases and Controls on the Basis of the Design or Analysis^e^** | **Ascertain-ment of Exposure^f^** | **Same Method of Ascertainment for Cases and Controls^g^** | **Non-Response Rate^h^** |  |
| Bollars [[5](#_ENREF_5)] | 2020 | a | a | a | a | c | a | a | a | 9 |
| Kaneko [[17](#_ENREF_17)] | 2021 | a | a | a | a | c | a | a | a | 9 |
| Savov [[37](#_ENREF_37)] | 2021 | a | a | a | a | c | a | a | a | 9 |

^a^ Possible assessments: a) yes, with independent validation (*); b) yes, eg, record linkage or based on self reports; c) no description.

^b^ Possible assessments: a) consecutive or obviously representative series of cases (*); b) potential for selection biases or not stated.

^c^ Possible assessments: a) community controls (*); b) hospital controls; c) no description.

^d^ Possible assessments: a) no history of disease (endpoint) (*); b) no description of source.

^e^ Possible assessments: a) study controls for most important factor (*); b) study controls for additional factor (*); c) study controls for most important and additional factor (**); d) no univariate or multivariate analyses or matching.

^f^ Possible assessments: a) secure record (eg surgical records) (*); b) structured interview where blind to case/control status (*); c) interview not blinded to case/control status; d) written self report or medical record only; e) no description.

^g^ Possible assessments: a) yes (*); b) no.

^h^ Possible assessments: a) same rate for both groups (*); b) non respondents described; c) rate different and no designation.

^i^ Sum of stars.

# References

1. Archer A, Coppolecchia A, Salem HS, Mont MA (2021) Lengths of Stay and Discharge Dispositions after Total Knee Arthroplasty: A Comparison of Robotic-Assisted and Manual Techniques. J Knee Surg.

2. Bendich I, Kapadia M, Alpaugh K, Diane A, Vigdorchik J, Westrich G (2021) Trends of Utilization and 90-Day Complication Rates for Computer-Assisted Navigation and Robotic Assistance for Total Knee Arthroplasty in the United States From 2010 to 2018. Arthroplasty Today 11:134-9.

3. Bhimani SJ, Bhimani R, Smith A, Eccles C, Smith L, Malkani A (2020) Robotic-assisted total knee arthroplasty demonstrates decreased postoperative pain and opioid usage compared to conventional total knee arthroplasty. Bone Jt Open 1:8-12.

4. Blum CL, Lepkowsky E, Hussein A, Wakelin EA, Plaskos C, Koenig JA (2021) Patient expectations and satisfaction in robotic-assisted total knee arthroplasty: a prospective two-year outcome study. Arch Orthop Trauma Surg 141:2155-64.

5. Bollars P, Boeckxstaens A, Mievis J, Kalaai S, Schotanus MGM, Janssen D (2020) Preliminary experience with an image-free handheld robot for total knee arthroplasty: 77 cases compared with a matched control group. Eur J Orthop Surg Traumatol 30:723-9.

6. Cho KJ, Seon JK, Jang WY, Park CG, Song EK (2019) Robotic versus conventional primary total knee arthroplasty: clinical and radiological long-term results with a minimum follow-up of ten years. Int Orthop 43:1345-54.

7. Cool CL, Jacofsky DJ, Seeger KA, Sodhi N, Mont MA (2019) A 90-day episode-of-care cost analysis of robotic-arm assisted total knee arthroplasty. J Comp Eff Res 8:327-36.

8. Cotter EJ, Wang J, Illgen RL (2022) Comparative Cost Analysis of Robotic-Assisted and Jig-Based Manual Primary Total Knee Arthroplasty. J Knee Surg 35:176-84.

9. Emara AK, Zhou G, Klika AK, Koroukian SM, Schiltz NK, Krebs VE, et al. (2021) Robotic-arm-assisted Knee Arthroplasty Associated With Favorable In-hospital Metrics and Exponentially Rising Adoption Compared With Manual Knee Arthroplasty. J Am Acad Orthop Surg 29:e1328-e42.

10. Fang CJ, Sun DC, Shaker JM, Talmo CT, Mattingly DA, Smith EL, et al. (2022) Total Knee Arthroplasty Hospital Costs by Time-Driven Activity-Based Costing: Robotic vs Conventional. Arthroplasty Today 13:43-7.

11. Greiner JJ, Wang JF, Mitchell J, Hetzel SJ, Lee EJ, Illgen RL (2020) Opioid Use in Robotic-Arm Assisted Total Knee Arthroplasty: A Comparison to Conventional Manual Total Knee Arthroplasty. Surg Technol Int 37:280-9.

12. Grosso MJ, Li WT, Hozack WJ, Sherman M, Parvizi J, Courtney PM (2022) Short-Term Outcomes Are Comparable between Robotic-Arm Assisted and Traditional Total Knee Arthroplasty. J Knee Surg 35:798-803.

13. Hamilton DA, Ononuju U, Chen C, Darwiche H, Nowak C (2021) Differences in Immediate Postoperative Outcomes Between Robotic-Assisted TKA and Conventional TKA. Arthroplasty Today 8:57-62.

14. Held MB, Gazgalis A, Neuwirth AL, Shah RP, Cooper HJ, Geller JA (2022) Imageless robotic-assisted total knee arthroplasty leads to similar 24-month WOMAC scores as compared to conventional total knee arthroplasty: a retrospective cohort study. Knee Surg Sports Traumatol Arthrosc 30:2631-8.

15. Held MB, Grosso MJ, Gazgalis A, Sarpong NO, Boddapati V, Neuwirth A, et al. (2021) Improved Compartment Balancing Using a Robot-Assisted Total Knee Arthroplasty. Arthroplasty Today 7:130-4.

16. Jeon SW, Kim KI, Song SJ (2019) Robot-Assisted Total Knee Arthroplasty Does Not Improve Long-Term Clinical and Radiologic Outcomes. J Arthroplasty 34:1656-61.

17. Kaneko T, Igarashi T, Takada K, Yoshizawa S, Ikegami H, Musha Y (2021) Robotic-assisted total knee arthroplasty improves the outlier of rotational alignment of the tibial prosthesis using 3DCT measurements. Knee 31:64-76.

18. Kayani B, Konan S, Tahmassebi J, Pietrzak JRT, Haddad FS (2018) Robotic-arm assisted total knee arthroplasty is associated with improved early functional recovery and reduced time to hospital discharge compared with conventional jig-based total knee arthroplasty: a prospective cohort study. Bone Joint J 100-b:930-7.

19. Kayani B, Konan S, Huq SS, Tahmassebi J, Haddad FS (2019) Robotic-arm assisted total knee arthroplasty has a learning curve of seven cases for integration into the surgical workflow but no learning curve effect for accuracy of implant positioning. Knee Surg Sports Traumatol Arthrosc 27:1132-41.

20. Kayani B, Tahmassebi J, Ayuob A, Konan S, Oussedik S, Haddad FS (2021) A prospective randomized controlled trial comparing the systemic inflammatory response in conventional jig-based total knee arthroplasty versus robotic-arm assisted total knee arthroplasty. Bone Joint J 103-B:113-22.

21. Khlopas A, Sodhi N, Hozack WJ, Chen AF, Mahoney OM, Kinsey T, et al. (2020) Patient-Reported Functional and Satisfaction Outcomes after Robotic-Arm-Assisted Total Knee Arthroplasty: Early Results of a Prospective Multicenter Investigation. J Knee Surg 33:685-90.

22. Kim Y-H, Yoon S-H, Park J-W (2020) Does Robotic-assisted TKA Result in Better Outcome Scores or Long-Term Survivorship Than Conventional TKA? A Randomized, Controlled Trial. Clin Orthop Relat Res 478:266-75.

23. King CA, Bradley AT, Jordan M, Wlodarski C, Tauchen A, Puri L (2022) Transitioning a Practice to Robotic Total Knee Arthroplasty Is Correlated with Favorable Short-Term Clinical Outcomes-A Single Surgeon Experience. J Knee Surg 35:78-82.

24. Liow MHL, Goh GS, Wong MK, Chin PL, Tay DK, Yeo SJ (2017) Robotic-assisted total knee arthroplasty may lead to improvement in quality-of-life measures: a 2-year follow-up of a prospective randomized trial. Knee Surg Sports Traumatol Arthrosc 25:2942-51.

25. Mahoney O, Kinsey T, Sodhi N, Mont MA, Chen AF, Orozco F, et al. (2022) Improved Component Placement Accuracy with Robotic-Arm Assisted Total Knee Arthroplasty. J Knee Surg 35:337-44.

26. Marchand RC, Sodhi N, Khlopas A, Sultan AA, Harwin SF, Malkani AL, et al. (2017) Patient Satisfaction Outcomes after Robotic Arm-Assisted Total Knee Arthroplasty: A Short-Term Evaluation. J Knee Surg 30:849-53.

27. Marchand RC, Sodhi N, Anis HK, Ehiorobo J, Newman JM, Taylor K, et al. (2019) One-Year Patient Outcomes for Robotic-Arm-Assisted versus Manual Total Knee Arthroplasty. J Knee Surg 32:1063-8.

28. Marchand KB, Ehiorobo J, Mathew KK, Marchand RC, Mont MA (2022) Learning Curve of Robotic-Assisted Total Knee Arthroplasty for a High-Volume Surgeon. J Knee Surg 35:409-15.

29. Marchand KB, Moody R, Scholl LY, Bhowmik-Stoker M, Taylor KB, Mont MA, et al. (2021) Results of Robotic-Assisted Versus Manual Total Knee Arthroplasty at 2-Year Follow-up. J Knee Surg.

30. Mitchell J, Wang J, Bukowski B, Greiner J, Wolford B, Oyer M, et al. (2021) Relative Clinical Outcomes Comparing Manual and Robotic-Assisted Total Knee Arthroplasty at Minimum 1-Year Follow-up. HSS J 17:267-73.

31. Mont MA, Cool C, Gregory D, Coppolecchia A, Sodhi N, Jacofsky DJ (2021) Health Care Utilization and Payer Cost Analysis of Robotic Arm Assisted Total Knee Arthroplasty at 30, 60, and 90 Days. J Knee Surg 34:328-37.

32. Naziri Q, Cusson BC, Chaudhri M, Shah NV, Sastry A (2019) Making the transition from traditional to robotic-arm assisted TKA: What to expect? A single-surgeon comparative-analysis of the first-40 consecutive cases. J Orthop 16:364-8.

33. Ofa SA, Ross BJ, Flick TR, Patel AH, Sherman WF (2020) Robotic Total Knee Arthroplasty vs Conventional Total Knee Arthroplasty: A Nationwide Database Study. Arthroplasty Today 6:1001.

34. Pelkowski JN, Wilke BK, Crowe MM, Sherman CE, Ortiguera CJ, Ledford CK (2020) Robotic-Assisted versus Manual Total Knee Arthroplasty in a Crossover Cohort: What Did Patients Prefer? Surg Technol Int 37:336-40.

35. Pierce J, Needham K, Adams C, Coppolecchia A, Lavernia C (2020) Robotic arm-assisted knee surgery: An economic analysis. Am J Manag Care 26:E205-E10.

36. Samuel LT, Cantrell WA, George JW, Higuera-Rueda CA, Kamath AF, Khlopas A, et al. (2021) Robotic Arm-Assisted versus Manual Total Knee Arthroplasty: A Propensity Score-Matched Analysis. J Knee Surg.

37. Savov P, Tuecking L-R, Windhagen H, Ehmig J, Ettinger M (2021) Imageless robotic handpiece-assisted total knee arthroplasty: a learning curve analysis of surgical time and alignment accuracy. Arch Orthop Trauma Surg 141:2119-28.

38. Shah R, Hyer JM, Tsilimigras D, Pawlik TM, Diaz A, Phieffer L, et al. (2021) Robotic total knee arthroplasty: A missed opportunity for cost savings in Bundled Payment for Care Improvement initiatives? Surgery (United States) 170:134-9.

39. Shaw JH, Lindsay-Rivera KG, Weir RM, Banka TR, Davis JJ, Buckley PJ (2021) Minimal Clinically Important Difference in Robotic-Assisted Total Knee Arthroplasty Versus Standard Manual Total Knee Arthroplasty. J Arthroplasty 36:S233-S41.

40. Smith AF, Eccles CJ, Bhimani SJ, Denehy KM, Bhimani RB, Smith LS, et al. (2021) Improved Patient Satisfaction following Robotic-Assisted Total Knee Arthroplasty. J Knee Surg 34:730-8.

41. Sodhi N, Khlopas A, Piuzzi NS, Sultan AA, Marchand RC, Malkani AL, et al. (2018) The Learning Curve Associated with Robotic Total Knee Arthroplasty. J Knee Surg 31:17-21.

42. Steffens D, Karunaratne S, McBride K, Gupta S, Horsley M, Fritsch B (2022) Implementation of robotic-assisted total knee arthroplasty in the public health system: a comparative cost analysis. Int Orthop 46:481-8.

43. Thiengwittayaporn S, Uthaitas P, Senwiruch C, Hongku N, Tunyasuwanakul R (2021) Imageless robotic-assisted total knee arthroplasty accurately restores the radiological alignment with a short learning curve: a randomized controlled trial. Int Orthop 45:2851-8.

44. Tompkins GS, Sypher KS, Li HF, Griffin TM, Duwelius PJ (2022) Robotic Versus Manual Total Knee Arthroplasty in High Volume Surgeons: A Comparison of Cost and Quality Metrics. J Arthroplasty 37:S782-s9.

45. Tompkins GS, Sypher KS, Griffin TM, Duwelius PD (2021) Can a Reduction in Revision Rates Make Robotic Total Knee Arthroplasty Cost Neutral With Manual Total Knee Arthroplasty at Ten-Year Follow-Up? An Episode Cost Analysis. Journal of Arthroplasty.

46. Vanlommel L, Neven E, Anderson MB, Bruckers L, Truijen J (2021) The initial learning curve for the ROSA Knee System can be achieved in 6-11 cases for operative time and has similar 90-day complication rates with improved implant alignment compared to manual instrumentation in total knee arthroplasty. J Exp Orthop 8:119.

47. Vermue H, Luyckx T, Winnock de Grave P, Ryckaert A, Cools AS, Himpe N, et al. (2022) Robot-assisted total knee arthroplasty is associated with a learning curve for surgical time but not for component alignment, limb alignment and gap balancing. Knee Surg Sports Traumatol Arthrosc 30:593-602.

48. Yang HY, Seon JK, Shin YJ, Lim HA, Song EK (2017) Robotic Total Knee Arthroplasty with a Cruciate-Retaining Implant: A 10-Year Follow-up Study. Clin Orthop Surg 9:169-76.

49. Zak SG, Yeroushalmi D, Tang A, Meftah M, Schnaser E, Schwarzkopf R (2021) The Use of Navigation or Robotic-Assisted Technology in Total Knee Arthroplasty Does Not Reduce Postoperative Pain. J Knee Surg.

50. Zhang J, Matzko CN, Sawires A, Ehiorobo JO, Mont MA, Hepinstall MS (2022) Adoption of Robotic-Arm-Assisted Total Knee Arthroplasty Is Associated with Decreased Use of Articular Constraint and Manipulation under Anesthesia Compared to a Manual Approach. J Knee Surg 35:849-57.
